# Non-associative Potentiation of Perisomatic Inhibition Alters the Temporal Coding of Neocortical Layer 5 Pyramidal Neurons

**DOI:** 10.1371/journal.pbio.1001903

**Published:** 2014-07-08

**Authors:** Joana Lourenço, Simone Pacioni, Nelson Rebola, Geeske M. van Woerden, Silvia Marinelli, David DiGregorio, Alberto Bacci

**Affiliations:** 1 European Brain Research Institute, Rome, Italy; 2 Sorbonne Universités UPMC Univ. Paris 06, UMR S 1127, Paris, France; 3 Inserm U 1127, Paris, France; 4 CNRS UMR 7225, Paris, France; 5 ICM- Institut du Cerveau et de la Moelle épinière, Paris, France; 6 CNRS UMR 3571, Paris, France; 7 Institut Pasteur, Unit of Dynamic Neuronal Imaging, Paris, France; The Salk Institute for Biological Studies, United States of America

## Abstract

In the neocortex, the coexistence of temporally locked excitation and inhibition governs complex network activity underlying cognitive functions, and is believed to be altered in several brain diseases. Here we show that this equilibrium can be unlocked by increased activity of layer 5 pyramidal neurons of the mouse neocortex. Somatic depolarization or short bursts of action potentials of layer 5 pyramidal neurons induced a selective long-term potentiation of GABAergic synapses (LTPi) without affecting glutamatergic inputs. Remarkably, LTPi was selective for perisomatic inhibition from parvalbumin basket cells, leaving dendritic inhibition intact. It relied on retrograde signaling of nitric oxide, which persistently altered presynaptic GABA release and diffused to inhibitory synapses impinging on adjacent pyramidal neurons. LTPi reduced the time window of synaptic summation and increased the temporal precision of spike generation. Thus, increases in single cortical pyramidal neuron activity can induce an interneuron-selective GABAergic plasticity effectively altering the computation of temporally coded information.

## Abstract 2

Long-term potentiation of inhibitory GABAergic transmission controls synaptic integration and action potential generation of specific neocortical neurons.

## Author Summary

The proper activity of cortical neurons (the brain cells responsible for memory and consciousness) relies on the precise integration of excitatory and inhibitory inputs. The excitation and inhibition (E/I) ratio has to remain constant both in time and strength to prevent neurological and psychiatric diseases. Fast inhibitory synaptic inputs to cortical pyramidal neurons originate from a rich diversity of GABAergic interneurons that operate a strict division of labor by differentially targeting precise regions of the pyramidal neurons. Here, we show that large pyramidal neurons of neocortical layer 5 can unlock the E/I ratio in response to their own activity. Excitatory activity of pyramidal neurons, in the form of membrane depolarization or trains of action potentials, induces a Ca^2+^-dependent mobilization of nitric oxide, which diffuses to inhibitory synapses and triggers a persistent enhancement of GABA release. Notably, this potentiation of inhibition is specific for synapses originating from parvalbumin (PV)-expressing interneurons that target mainly the perisomatic region of pyramidal neurons. Long-term potentiation of perisomatic inhibition, in turn, changes the ability of pyramidal neurons to integrate excitatory inputs as well as the temporal properties of their own action potential output. Selective plasticity of perisomatic inhibition can thus play a crucial role in cortical activity, such as sensory processing and integration.

## Introduction

In the cerebral cortex, fast GABAergic inhibition is tightly coupled to excitation both temporally and in strength. This constant balance of opposing forces is necessary for the correct development of cortical sensory receptive fields [1] and allows for the generation and tuning of cortical network activity underlying cognitive functions and complex behaviors [2]. Indeed, it has been proposed that alterations of this equilibrium result in devastating neurological and/or psychiatric diseases, such as epilepsy, schizophrenia, and autism [3]. Studies have shown that dynamic cellular mechanism are capable of compensating changes in synaptic excitation in order to maintain a particular excitation-to-inhibition (E/I) ratio intact, for example, either by weakening of feed-forward inhibition [4] or persistently enhancing inhibitory neurons' excitability [5]. Nevertheless, perturbations in the E/I balance can play a key role in sensory learning and receptive field reorganization [6],[7], suggesting it may be necessary to unlock the restrictive gate on the E/I balance. However, no such cellular mechanisms have been demonstrated. Moreover, the E/I ratio is remarkably different across cortical layers, resulting in layer-specific suppression or augmentation of pyramidal neuron output in response to sustained input activation [8]. Thus, E/I ratios can be state-dependent and regulated according to computational requirements of specific microcircuit pathways.

In principle, short- and long-term forms of synaptic plasticity of either inhibitory or excitatory neurotransmission could be responsible for dynamically altering the E/I ratio of specific cortical networks. This is especially true for cortical GABAergic synapses as they originate from a rich diversity of interneuron types [9],[10], which may differentially modulate the excitatory information flow along the dendro-somatic axis of pyramidal neurons.

In this context, alteration of the E/I ratio might have important and specific consequences in input–output transformations of pyramidal neurons and their ability to integrate and relay different salient features of sensory information. Although the E/I ratio is usually referred to as a “global” balance, it is not known whether specific inhibitory circuits can induce region-specific unlocking of this equilibrium.

Interestingly, we have previously found that layer 2/3 pyramidal neurons can self-tune their excitability and inhibitory synaptic strength solely in response to their own activity [11]. Whether this mechanism can alter the E/I balance is not known.

Here we show that in contrast to layer 2/3, single layer 5 pyramidal neurons' activity alone can alter E/I balance by inducing long-term potentiation of perisomatic inhibitory GABAergic transmission (LTPi) while leaving the strength of glutamatergic inputs unchanged. Moreover, this plasticity is specific for inhibition originating from parvalbumin (PV)-positive basket cells and not somatostatin (SST)-expressing interneurons, which target distal dendrites. Physiological burst-firing patterns of pyramidal neurons are sufficient to induce retrograde signaling of nitric oxide (NO), which increases GABA release from NO-sensitive PV presynaptic terminals. This non-associative potentiation of perisomatic GABAergic synapses results in an efficient layer 5 alteration of the balance between excitation and inhibition, reducing firing probability and, importantly, markedly sharpening the time window of synaptic integration. This activity-dependent auto-modulation of layer 5 neocortical pyramidal neurons is ideally suited to enhance sparseness and improve the precision of time-coded information processing in a region-specific manner.

## Results

### Postsynaptic Depolarization of Layer V Pyramidal Neurons Selectively Potentiates GABAergic Inputs

**Figure 1:**
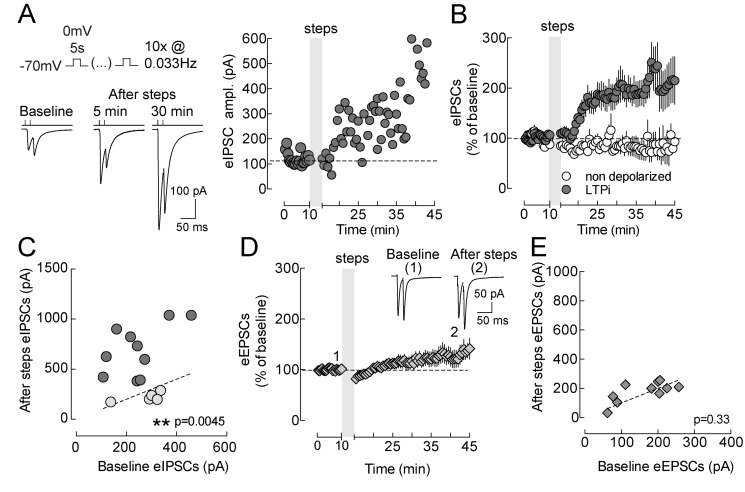
Postsynaptic depolarization of layer V pyramidal neurons selectively potentiates GABAergic inputs. (A, Left) Representative voltage-clamp traces of monosynaptic extracellularly evoked pairs of IPSCs evoked at 50 Hz, in the continuous presence of ionotropic AMPA/Kainate glutamate receptor antagonists (DNQX, 10 µM), before and at two time points after 10 intracellular depolarizations to 0 mV (5 s, delivered every 30 s; schematized on top). Each trace is the average of 10 sweeps. Shown are responses to paired-pulse stimulations. Postsynaptic depolarizations resulted in a strong increase of eIPSC amplitude. (A, Right) Time course of IPSCs (the first of the paired-pulse responses, averaged in 30 s bins) of the cell of (A), displaying a clear LTPi. The shaded area refers to postsynaptic depolarizing steps. (B) Average time course of relative IPSC changes in cells subject to somatic depolarization (solid circles) and nondepolarized cells (open circles). The shaded area refers to postsynaptic depolarizing steps. (C) Plot of individual eIPSC amplitudes before (*x*-axes) versus 20 min after postsynaptic depolarization (steps) (*y*-axes). The majority of layer 5 pyramidal neurons expressed a long-term change in eIPSC amplitude, which we designated as LTPi (grey circles). A small percentage of the cells do not express LTPi (open circles). Dotted line indicates unitary values (no change). Grey symbols and white symbols refer to pyramidal neurons that did and did not express LTPi, respectively. (D) Average time course evoked glutamatergic EPSCs, isolated pharmacologically in the continuous presence of gabazine. The same depolarizing steps did not cause any significant change in EPSC amplitudes. Inset shows representative EPSC traces taken immediately before and 30 min after induction of LTPi. (D). (E) eEPSC amplitudes after postsynaptic depolarizations plotted as a function of their baseline values. Dotted line indicates unitary values (no change). Numbers (1 and 2) refer to times of trace illustration.

We examined whether layer 5 pyramidal neurons can modulate the strength of GABAergic synapses by postsynaptic depolarization similarly to layer 2/3 pyramidal neurons [11], and if also glutamatergic transmission could be altered by postynaptic depolarization protocols. Inhibitory postsynaptic currents (IPSCs) onto layer 5 pyramidal neurons were evoked by extracellularly stimulating their perisomatic afferents, in the continuous presence of the ionotropic glutamate receptor antagonist DNQX (10 µm). Surprisingly, in contrast to layer 2/3 pyramidal neurons [11], which responded to repeated somatic depolarizing steps with LTDi, a similar protocol (ten 5-s long steps to 0 mV, repeated every 30 s from a holding potential of −70 mV) induced a robust increase in the amplitude of eIPSCs onto layer 5 pyramidal neurons. LTPi persisted for >30 min (eIPSCs baseline, 260.1±24.03 pA; eIPSCs 20 min after steps, 517.4±77.50 pA, *n* = 16, *p* = 0.0045, paired *t* test; Figure 1A,B; normalized changes of eIPSCs, see Materials and Methods; ΔeIPSCs amplitude = 129.0±40.7%, Figure S1A), and interestingly, it occurred in the absence of any presynaptic stimulation during somatic depolarizing steps (non-associative LTPi). An increase in eIPSCs amplitude of at least 50% of eIPSCs baseline amplitude was present in 71 out of 101 (control or vehicle) tested layer 5 pyramidal neurons (71.7%; e.g. Figure 1C). Importantly, LTPi-inducing protocols failed to induce long-term plasticity of glutamatergic excitatory synaptic responses, which were isolated in the continuous presence of the GABA_A_R antagonist gabazine (baseline, 161.6±22.08 pA; after steps, 178±21.86 pA, *n* = 10, *p* = 0.3355, paired *t* test; Figure 1D and 1E). This potentiation of inhibitory but not excitatory synapses likely results in an unbalanced E/I ratio (see below).

### Postsynaptic Action Potential (AP) Firing Efficiently Induces LTPi

**Figure 2:**
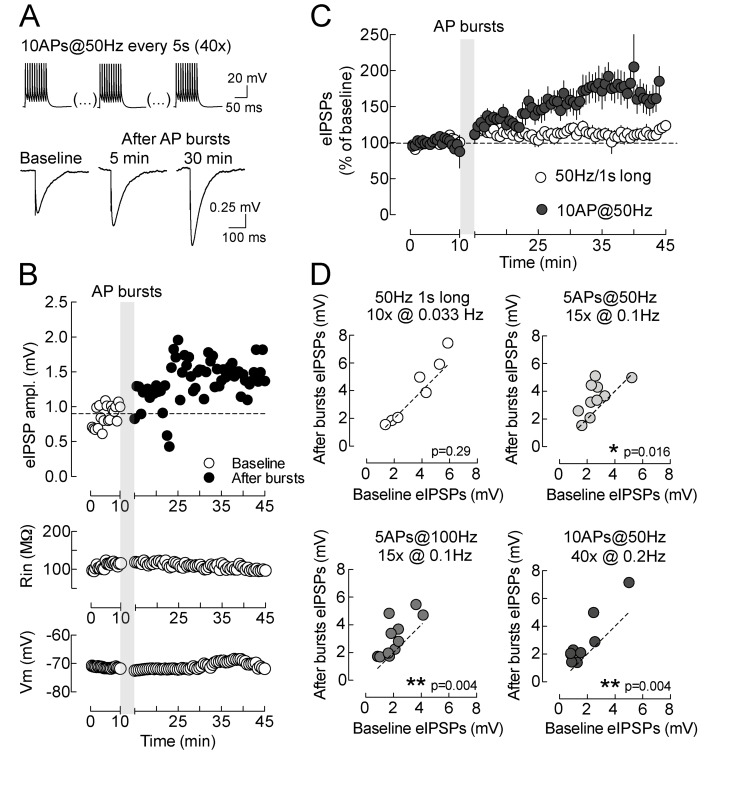
Postsynaptic AP firing efficiently induces LTPi. (A) Representative current-clamp traces of eIPSPs in the presence of intracellular physiological [Cl^−^] in continuous presence of DNQX (10 µM). A clear LTPi is observed 30 min after postsynaptic AP bursts (Top). (B) Time course of IPSPs (binned in 30-s intervals) in the neuron of (A) before (open circles) and after (solid circles) postsynaptic AP bursts. Input resistance (Rin, Middle) and resting membrane potential (Vm, Bottom) remained stable throughout the experiment. (C) Population time courses of normalized IPSPs in neurons firing long spike trains (open circles), showing no LTPi, and in neurons firing brief AP bursts (solid circles) expressing LTPi. (D) Plots of individual eIPSPs amplitude before (*x*-axes) versus 20 min after AP bursts (*y*-axes). LTPi could be induced by different burst firing paradigms (solid circles) but not long-lasting firing (open circles). Data refer to single values and/or mean ± SEM. *p<0.05; **p<0.001.

Although layer 5 pyramidal neurons fire rather irregularly during awake asynchronous states, they commonly display high-frequency (>100 Hz) burst firing depending on the behavioral state of the animal [12],[13]. We therefore tested if short bursts of APs (induced by somatic current injections) could increase GABAergic synaptic strength, similarly to somatic depolarizations in voltage clamp. We recorded pharmacologically isolated evoked inhibitory postsynaptic potentials (eIPSPs) in layer 5 pyramidal neurons, in current-clamp mode with physiological intracellular chloride (see Materials and Methods). Repeated bursts of APs (5–10 spikes, at 50 or 100 Hz; Figure 2D) efficiently increased GABAergic transmission onto layer 5 pyramidal neurons [1.8±0.4 versus 2.92±0.63 mV, baseline versus after 20 min after AP bursts (10 AP at 50 Hz), respectively, *n* = 9, *p* = 0.004, Wilcoxon signed rank test; Figure 2A–D]. Interestingly, repeated 1-s-long AP bursts at 50 Hz failed to induce GABAergic plasticity (3.95±0.86 versus 3.53±0.66 mV, baseline versus after 20 min after AP bursts, respectively; *n* = 7, *p* = 0.29 Wilcoxon signed rank test; Figure 2C and 2D). These experiments indicate that LTPi can be induced in current clamp by short postsynaptic bursts of APs alone. Taken together, these results show a non-associative form of LTP of inhibitory synapses onto layer 5 pyramidal neurons: inhibition is increased by postsynaptic activity without the requirement of concomitant presynaptic stimulation.

### LTPi Is Selectively Expressed at Perisomatic GABAergic Synapses from PV Cells

**Figure 3:**
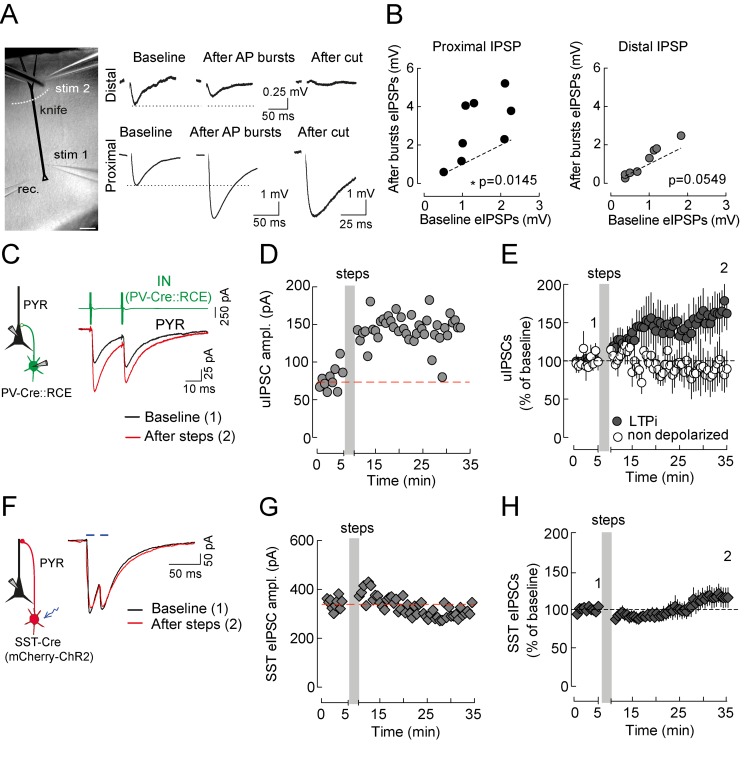
LTPi is selectively expressed at perisomatic GABAergic synapses from PV cell. (A, Left) Micrograph showing recording and stimulating configurations. The dashed white line schematizes the cut by a fourth broken glass pipette. (A, Right Top) Representative current-clamp traces of distally evoked eIPSPs before, after AP bursts (5 APs at 100 Hz, repeated 15×) and after cut. (A, Right Bottom) Representative traces of proximally evoked eIPSPs in the same conditions. (B) Plots of proximal (Left) and distal (Right) IPSP amplitudes in control versus 20 min after LTPi-inducing AP bursts. (C, Left) Schematic of simultaneous paired recordings from a presynaptic PV basket cell and a postsynaptic layer 5 pyramidal neuron. PV cells were identified as expressing EGFP in PV-Cre::RCE mice (see Materials and Methods) [18]. (C, Right) Action currents in voltage clamp (green) in the presynaptic interneuron (IN) trigger uIPSCs in the postsynaptic pyramidal neuron (PYR). Black trace, before the depolarizing steps; red trace, after induction of d-LTPi. (D) Time course of the cell of (C), showing a persistent increase of uIPSCs after postsynaptic depolarizing steps (grey area). (E) Population data of paired recordings, showing LTPi when the postsynaptic cell was depolarized (filled symbols) and absence of plasticity when pyramidal neurons were not depolarized. Data are single values and/or mean ± SEM. *p<0.05. (F) Schematic of optogenetic activation of SST-positive interneurons. ChR2 was co-expressed with mCherry in SST-cre mice using viral vectors (see Materials and Methods) [20]. (F, Right) Brief (2 ms) flashes of 470 nm light (blue bars) induced a GABAergic current that was insensitive to LTPi-inducing protocols (black trace, control; red trace, after postsynaptic depolarizing steps). (G) Time course of the cell of (F), showing lack of persistent increase of SST-mediated population IPSCs after postsynaptic depolarizing steps (grey area). (H) Population data of SST-mediated population IPSCs, showing lack of LTPi when the postsynaptic cell was depolarized. Numbers (1 and 2) refer to times of trace illustration.

In cortical structures, including the neocortex, perisomatic and dendritic GABAergic inhibition is provided by distinct interneuron types [9],[14]. We therefore sought to identify if LTPi was a general property of GABAergic synapses or if it was present at a specific inhibitory circuit. First, perisomatic and dendritic GABAergic synapses were evoked [in the continuous presence of DNQX (10 µM)] in the same neuron by placing two stimulation electrodes near pyramidal neurons' cell bodies (proximal, perisomatic stimulation) and at a distal (∼400 µm) dendritic location, respectively (Figure 3A). The integrity of dendrites was confirmed by visual inspection under IR-DIC video microscopy. In some cases, neurons were filled with the fluorescent dye Alexa 594 (20 µM) or with neurobiotin for post hoc histological reconstructions (unpublished data).

Perisomatic IPSPs could be reliably potentiated by repeated bursts of 5 APs at 100 Hz (proximal baseline, 1.42±0.23 mV; proximal after AP bursts, 2.9±0.57 mV, *n* = 8, *p* = 0.0145, paired *t* test; Figure 3A and 3B). Layer 5 pyramidal cells were depolarized using AP trains in current clamp and in physiological chloride, as described in Figure 2. Interestingly, distal IPSPs were unaffected by the same postsynaptic firing protocol (distal baseline, 1.149±0.28 mV; distal after AP bursts, 0.88±0.17, *n* = 8, *p* = 0.0549, paired *t* test; Figure 3A,B). To confirm synaptic activation of dendritic and perisomatic inhibition, in some experiments we gently cut pyramidal neuron dendrites using a knife pipette, at the end of experiments. This procedure resulted in the disappearance of distally evoked IPSPs, leaving perisomatic responses unaltered (Figure 3A).

In the neocortex, perisomatic and dendritic inhibition are provided by different interneuron classes [9],[15],[16]. We tested plasticity of inhibition originating from PV-positive, fast-spiking (FS) basket cells, and SST-positive interneurons. The former target the perisomatic region of pyramidal neurons, whereas the latter target the distal portion of their apical dendrites [15],[17]. To identify GABAergic transmission originating from PV interneurons, we used paired recordings between PV cells and pyramidal neurons (using PV-Cre::RCE mice, Figure 3C–E; see Materials and Methods [18],[19]). Conversely, to selectively activate SST-cell IPSCs, we expressed the light-activated channel channelrhodopsin 2 (ChR2) using viral vectors in SST-Cre mice (see Materials and Methods [20]). Optogenetic activation of SST interneurons invariably produced a response that was abolished by gabazine and had a relatively slow rise time (2.9±0.1 ms, *n* = 9; Figures S2D and S3C,D), as compared to optogenetically evoked IPSCs from PV interneurons (2.0±0.1 ms; *n* = 6, *p*<0.05, Mann–Whitney test; Figure S3C–D) and consistent with dendritic electrotonic filtering. A residual, minimal inward light-induced current was present in gabazine when both SST and PV neurons were photostimulated (Figures S2D and S3E). This residual current was abolished by 0.5 µM TTX (Figure S3E) as previously reported [21].

In paired recordings between PV cells and pyramidal neurons, repeated depolarizing steps of postsynaptic pyramidal neurons potentiated unitary IPSCs (uIPSCs) in 7 out of 10 pairs (ΔuIPSC LTPi, 59.18±24.63%, *n* = 10, *p* = 0.0371, Wilcoxon signed rank test; Figure 3D–E). Importantly, non-depolarized PV–pyramidal neuron pairs showed no significant change over time (−13.18±19.15%, *n* = 5, *p*>0.05, Wilcoxon signed rank test; Figure 3E). In contrast, LTPi-inducing stimuli failed to trigger plasticity of SST-cell IPSCs, induced by brief (2 ms) flashes of blue light (λ = 470 nm) (baseline, 230.1±46.7; after steps, 239.9±47.86 pA, *n* = 9, *p* = 0.57, paired *t* test; Figure 3F–H).

Altogether, these results indicate that LTPi is interneuron-selective, as it affects perisomatic GABAergic synapses from PV basket cells, leaving dendritic inhibition from SST interneurons intact.

### LTPi Is Expressed Presynaptically

**Figure 4:**
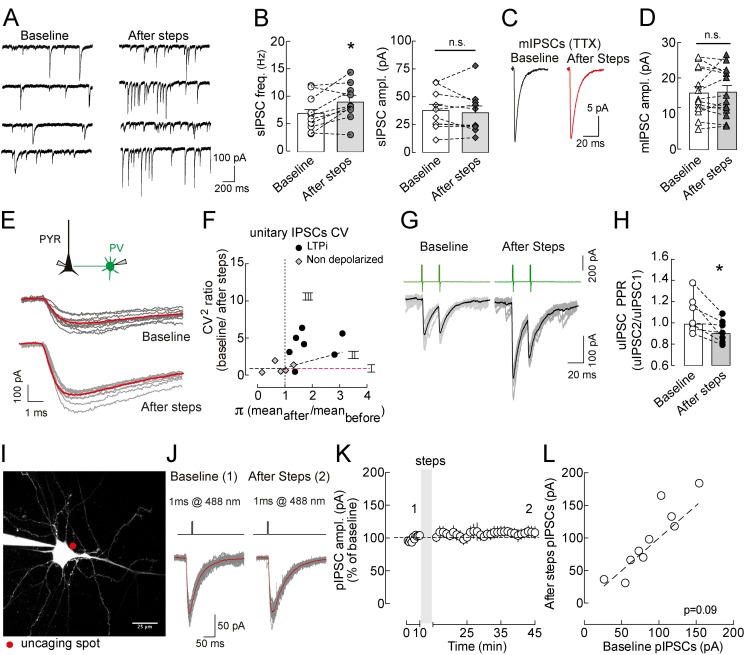
LTPi is expressed presynaptically. (A) Representative traces of spontaneous IPSCs (sIPSCs) during baseline and after LTPi induction. (B) Summary graph of sIPSC frequency (Left) and amplitude (Right) before (white bar, open circles) and after steps (grey bar, solid circles). Data are values from single cells and/or mean ± SEM. *p<0.05 as compared to baseline. (C) Average representative traces of mIPSCs recorded in the continuous presence of tetrodotoxin (TTX, 500 nM) before (black trace) and after (red trace) depolarizing steps. Each trace is the average of ∼100 single quantal events. (D) Population plot of mIPSC amplitudes before (empty symbols) and after depolarizing steps (filled symbols). n.s., not statistically significant (*p*>0.05). (E) Representative traces of PV basket cell–pyramidal neuron uIPSCs before and after LTPi; grey traces are overlapped single-sweep responses. Note the decrease in peak amplitude fluctuations of single responses during LTPi. This is associated with an increase of the mean response (red traces). (F) Analysis of the squared coefficients of variations of PV cell–pyramidal neuron uIPSCs (CV^2^1/CV^2^2) as described by Faber and Korn [59]. The numbers 1 and 2 refer to baseline and after depolarization values, respectively. According to this analysis, CV^2^ values on the horizontal line (I) reflect a postsynaptic potentiation, whereas cells in region II (above the diagonal linear fit line) showed a presynaptic LTPi expression. Values in region III refer to P neurons with a mixed pre- and postsynaptic LTPi expression. Overall, apart from few exceptions, LTPi had a presynaptic expression locus (above region II). Cells that did not express LTPi (grey diamonds) did not show IPSC CV^2^ changes compatible with synaptic potentiation. (G) Representative traces of two PV cell–pyramidal neuron uIPSCs evoked at 20-ms intervals, before (Left) and 20 min after (Right) LTPi. Grey traces are overlapped single sweeps; black lines are averaged traces. (H) Population data of PPR in baseline (white bar, open circles) and 20 min after depolarization (grey bar, solid circles). Note a decrease in PPR after the steps, indicating a change (an increase) in the probability of GABA release. Data are single values and/or mean ± SEM. **p<0.01 as compared to baseline. Further analyses are illustrated in Figure S1. (I) 2PSLM image (maximal intensity projection) of a layer 5 pyramidal neuron loaded with Alexa 594 (20 µM). The red dot shows the size (∼5 µm) and location of the uncaging spot on the perisomatic region. (J) 1 ms, 488 nm laser pulse elicited pIPSC (individual traces in grey and average in red) before and in the time window between 20 and 30 min after depolarizing steps. (K) Population data show no change in pIPSC amplitude after steps. (L) Correlation plot of pIPSC amplitudes in control versus 20 min after LTPi-inducing depolarizing steps.

Previous results indicated that postsynaptic depolarization of layer 5 visual cortical pyramidal neurons from hyperpolarized membrane potential (−90 mV) can induce long-term plasticity of GABAergic neurotransmission due to alterations of postsynaptic trafficking of GABA_A_Rs [22]. On the other hand, a recent study reported a non-Hebbian (i.e., non-associative) presynaptic form of GABAergic plasticity in the thalamus [23]. Therefore we decided to investigate the locus of expression of the LTPi described here. We found that LTPi (10 out of 16 cells, Figure 1C and Figure S1A) was accompanied by a significant increase in the frequency of spontaneous (s)IPSCs (baseline, 6.9±0.99 Hz; after steps, 8.9±1.04 Hz, *n* = 10, *p* = 0.0135, paired *t* test; Figure 4A,B, left panel) with no changes in their amplitudes (baseline, 37.66±5.5 pA; after steps, 35.74±5.9 pA, *n* = 10, *p* = 0.49, paired *t* test; Figure 4B, right panel). If LTPi resulted from increased GABA_A_R function at postsynaptic sites, a change in quantal synaptic event amplitudes would be apparent. In fact, amplitudes of miniature (m)IPSCs (recorded in 0.5 µM TTX) were unchanged by somatic LTPi-inducing depolarizing steps (baseline, 15.63±1.8 pA; after steps, 15.90±1.8 pA, *n* = 10, *p* = 0.75, paired *t* test; Figure 4C). Conversely, similarly to sIPSCs, mIPSC frequency also increased in response to LTPi-inducing depolarizing steps (13.29±3.8 versus 19.39±5.9 Hz, baseline versus 20 min after steps; *n* = 10, *p*<0.05, paired *t* test; Figure 4D). Importantly, mIPSCs had very fast rise times (<1 ms; Figure S1D), indicating that inhibitory quantal events were mostly perisomatic, as suggested by much faster IPSC rise times from PV as compared to SST cells (*p*<0.01; Figure S3C–D). No change of rise-time distribution was observed after LTPi-inducing stimuli (Figure S1D).

Accordingly, coefficient of variation (CV) analysis of uIPSCs obtained from connected PV-basket cells and pyramidal neurons revealed that five out of seven pairs exhibiting LTPi had a purely presynaptic locus of expression (Figure 4E,F). Moreover, the ratio of PV-cell–induced uIPSCs elicited at a short time interval (20 ms, paired-pulse ratio, or PPR) significantly decreased after LTPi (baseline, 1.1±0.06; after steps, 0.9±0.03, *n* = 7, *p* = 0.01, Wilcoxon signed rank test; Figure 4G,H). Also extracellularly evoked IPSCs increased their CV and decreased their PPR, following LTPi (Figure S1B,C,D), consistent with a presynaptic locus of plasticity. Importantly, these parameters remained unchanged in cells that did not express LTPi (Figure S1B and C).

To examine whether postsynaptic GABAergic plasticity also contributes to LTPi [22], we performed single photon photolysis of the caged GABA compound Rubi–GABA (20 µM) before and after LTPi, using a 5 µm laser spot (λ = 488 nm) positioned at the perisomatic region of layer 5 pyramidal neuron. Photolysis-evoked IPSCs (pIPSCs) were elicited with 1 ms laser pulses, producing baseline current amplitudes ranging between 25 and 160 pA. Following the same somatic depolarization used to induce LTPi, we did not detect alterations in the amplitude of pIPSCs (85.22±10.9 pA baseline and 97.92±14.73 pA after step depolarization, *n* = 11; *p*>0.05, paired *t* test; Figure 4I–L), ruling out a postsynaptic locus of LTPi.

Taken together, these results show that LTP of perisomatic inhibitory synapses is expressed primarily presynaptically.

### Elevation of Postsynaptic Calcium Through L-Type Ca^2+^ Channels Is Necessary to Induce LTPi

**Figure 5:**
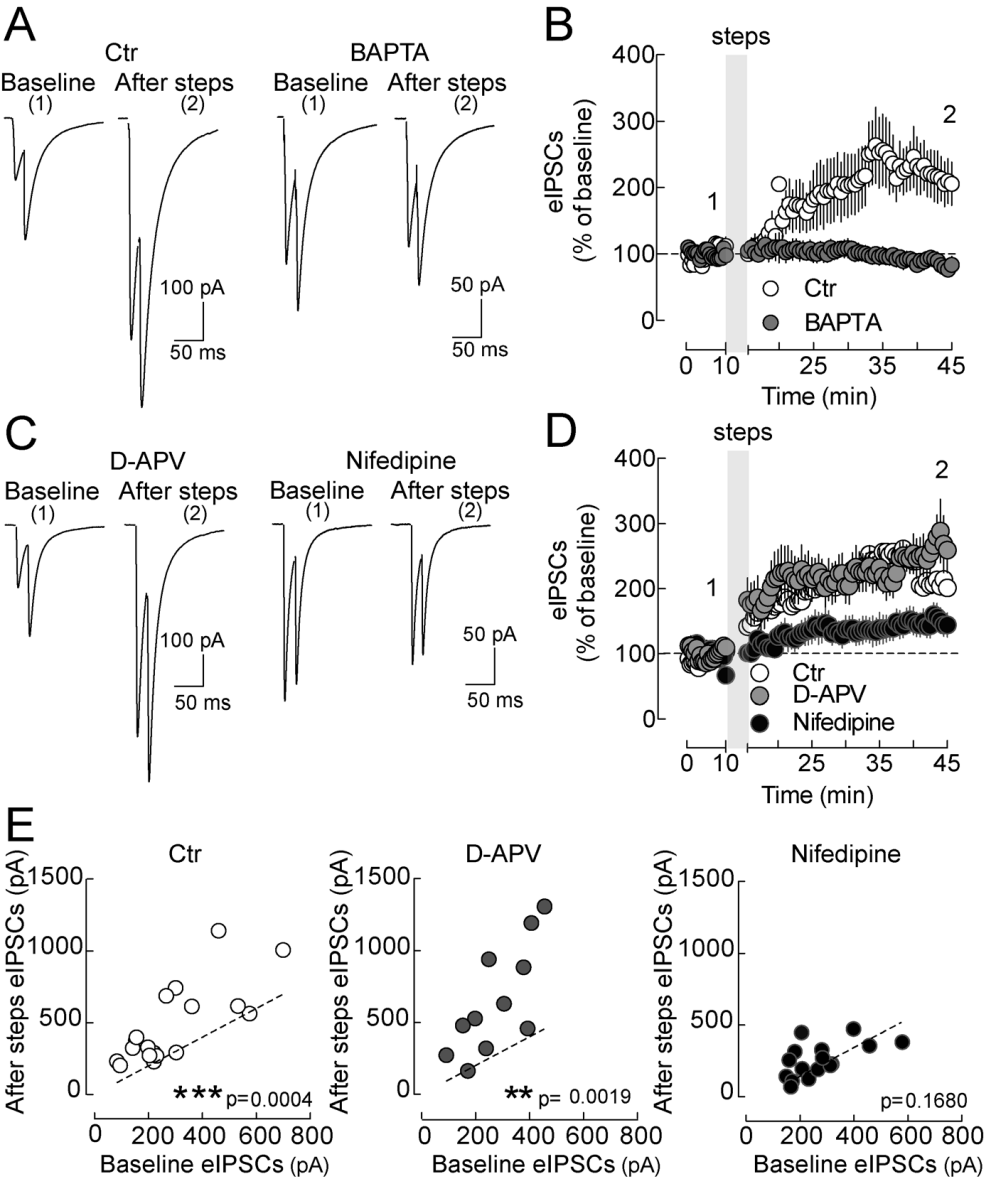
Elevation of postsynaptic calcium through L-type Ca^2+^ channels is necessary to induce LTPi. (A) Representative eIPSC traces recorded from two pyramidal neurons before (baseline) and 20 min after LTPi-inducing depolarizing steps. The left neuron was intracellularly perfused with 0.2 mM EGTA (Ctr), the right neuron with 20 mM BAPTA. Note that in the presence of the fast Ca^2+^ chelator LTPi could not be induced. (B) Population time courses of normalized IPSCs in the two conditions of (A). (C, Left) Representative eIPSC traces recorded from a pyramidal neuron expressing LTPi in the presence of the NMDA receptor antagonist (D-APV, 50 µM). (C, Right) Representative eIPSC traces of another pyramidal neuron, in which LTPi was prevented by the L-type Ca^2+^ channel blocker nifedipine (10 µM). (D) Population data plots of the two pharmacological applications of (C). (E) Plots of individual eIPSC amplitudes before (*x*-axes) versus 20 min after depolarizing steps (*y*-axes) in control (ctr), D-APV, and nifedipine. In (C–E), white, grey, and black symbols refer to control, D-APV, and nifedipine, respectively. Data are single values and/or mean ± SEM. **p<0.001; ***p<0.0001. Further analyses are illustrated in Figure S4A. Numbers 1 and 2 refer to times of trace illustration.

What are the cellular mechanisms underlying LTPi? Elevation of postsynaptic calcium concentration ([Ca^2+^]) is often involved in GABAergic synaptic plasticity [24]. To prevent postsynaptic [Ca^2+^] elevations in pyramidal neurons, we included the Ca^2+^chelator 1,2-bis-(o-aminophenoxy)-ethane-*N,N,N′,N′*-tetraaceticacid, tetraacetoxymethyl ester (BAPTA, 20 mM) in the intracellular pipette solution. In this condition, LTPi was prevented (IPSCs, 289.7±26.69 versus 243.0±32.64 pA, before versus 20 min after depolarizing steps, respectively; *n* = 9, *p* = 0.14, paired *t* test; Figure 5A,B and Figure S4A). Importantly, LTPi was present in control conditions, even when induced after up to 20 min of intracellular dialysis, following patch rupture (IPSCs, 289.3±43.93 versus 608.6±115.5 pA, before versus 20 min after depolarizing steps, respectively; *n* = 9, *p* = 0.0034, paired *t* test; Figure 5A,B and Figure S4A).

To confirm that intracellular Ca^2+^ elevations is required for the strengthening of inhibitory synapses originating from PV cells, we expressed ChR2 in PV cells and elicited IPSCs originating from this cell type selectively. We confirmed LTPi following photostimulation in control conditions (*p*<0.05; *n* = 6 Wilcoxon signed rank; Figure S3A–B), similarly to results illustrated in Figure 3C–E. Importantly, intracellular perfusion of BAPTA completely abolished LTPi (Figure S3A–B; *p*>0.05, *n* = 4) similarly to results shown in Figure 5A–B. In addition, these experiments confirm that photostimulated IPSCs can undergo LTPi.

Voltage-gated Ca^2+^ channels (VGCCs) and ionotropic glutamate NMDA receptors are efficient sources of postsynaptic Ca^2+^, classically involved in synaptic plasticity. We found that the L-type Ca^2+^ channel blocker nifedipine (10 µM) prevented LTPi (IPSCs, 240.2±30.02 versus 280.5±29.92 pA, before versus 20 min after depolarizing steps, respectively; *n* = 16, *p* = 0.168, paired *t* test; Figure 5C–E), whereas blockade of NMDARs with D-APV (50 µM) had no effect on this form of GABAergic plasticity (IPSCs, 277.3±36.02 versus 652.9±114.3 pA, before versus after 20 min after depolarizing steps, respectively; *n* = 11, *p* = 0.0019, paired *t* test; Figure 5C–E). Overall, these data show that postsynaptic Ca^2+^ signaling via L-type Ca^2+^ channels is important for LTPi induction.

### LTPi Requires NO-Sensitive Guanylyl Cyclase (NO-Sensitive GC) Signaling

**Figure 6:**
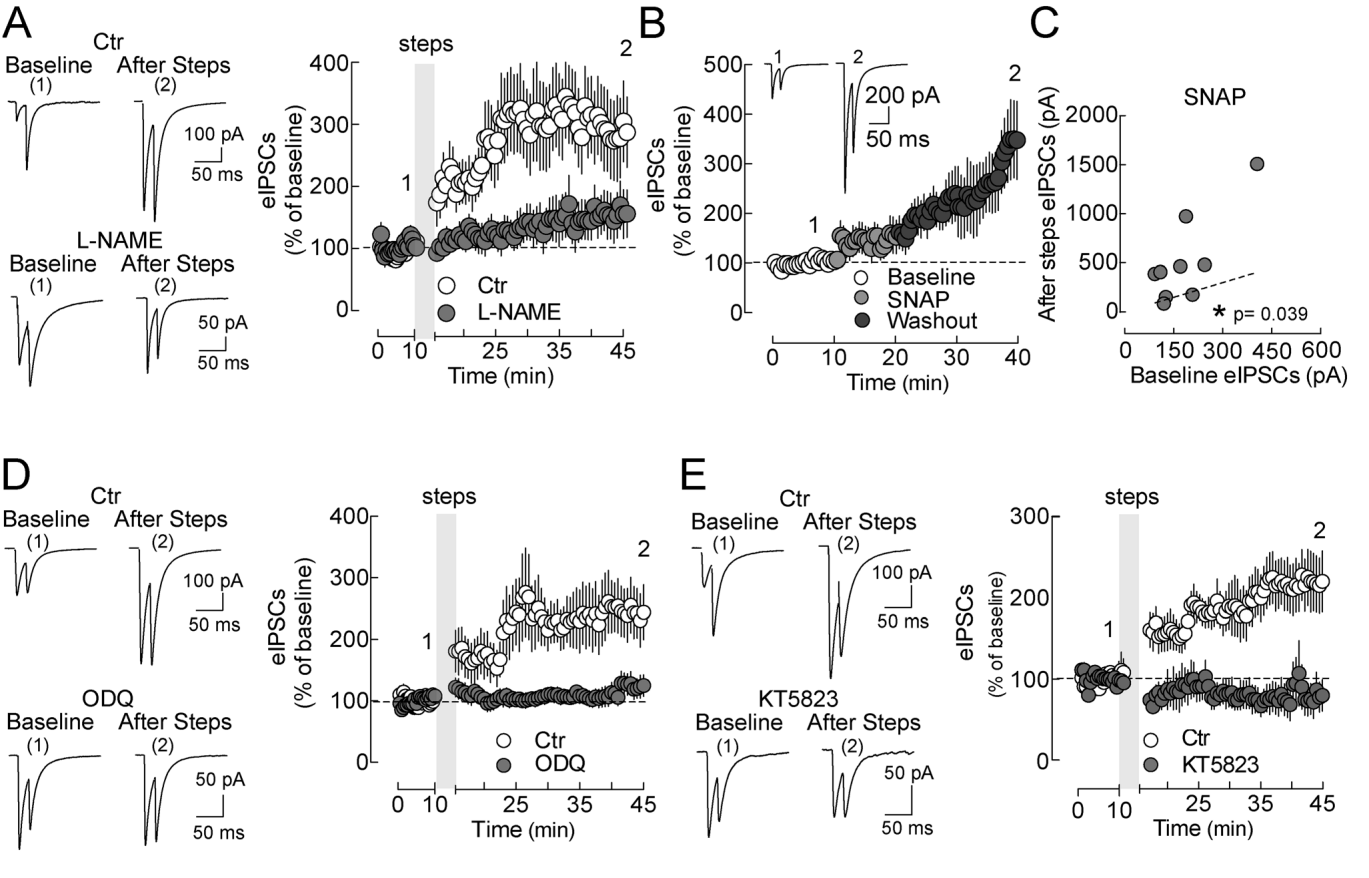
LTPi requires NO-sensitive GC signaling. (A, Left) representative eIPSC traces of a control pyramidal neuron (ctr; Top) expressing LTPi and another in the continuous presence of a broad NOS inhibitor (L-NAME, 100 µM; Bottom). (A, Right) Population data plots of the experiment of (A). Note that L-NAME prevented LTPi induction. (B) Time course of perisomatic IPSCs recorded from layer 5 pyramidal neurons in the presence of the NO donor SNAP (200 µM for 10 min) and the continuous presence of the nonselective phosphodiesterase inhibitor IBMX (200 µM). White, light grey, and grey symbols refer to control, SNAP, and washout periods, respectively. The inset illustrates representative IPSC traces at the two indicated time points (1 and 2). (C) Plot of individual eIPSCs amplitude before (*x*-axes) versus 20 min after SNAP application (*y*-axes). (D, Top) Representative eIPSC traces of a control pyramidal neuron (ctr; perfused with drug vehicle DMSO) expressing LTPi. (D, Bottom) Representative traces of other pyramidal neurons in the presence of the NO-sensitive GC inhibitor ODQ (10 µM). (D, Right) Population data plots showing LTPi blockade by ODQ. (E, Top) Representative eIPSC traces of a control pyramidal neuron (ctr) expressing LTPi. (E, Bottom) Representative traces of other pyramidal neurons in the presence of the PKG inhibitor KT5823 (500 nM). (E, Right) Population data plots showing LTPi blockade by KT5823. Data are single values and/or mean ± SEM. *p<0.05, as compared to raw values of eIPSCs during baseline. Further analyses are illustrated in Figure S4B–D. Numbers 1 and 2 refer to times of trace illustration.

Ca^2+^-dependent postsynaptic induction of persistent changes of presynaptic GABA release suggests the involvement of retrograde synaptic signaling. Two major molecular messengers have been indicated as responsible for retrograde synaptic signaling and plasticity: endocannabinoids and NO [11],[23],[25]. We found that CB1 blockade by AM-251 (2 µM) was ineffective in preventing LTPi (Figure S4B). However, when NO production was prevented by preincubation and constant perfusion of cortical slices with the general NO synthase inhibitor Nφ-nitro-L-arginine methyl ester (L-NAME, 100 µM), LTPi was blocked (IPSCs, 337.3±43.38 versus 473.9±115.2 pA before versus 20 min after depolarizing steps, *n* = 11, *p* = 0.22, paired *t* test; Figure 6A and Figure S4B). Importantly, LTPi could be reliably induced in interleaved control experiments, incubating slices with the L-NAME vehicle (IPSCs, 257.0±43.38 versus 660.2±119.7 pA, before versus 20 min after depolarizing steps, *n* = 15, *p* = 0.001, paired *t* test; Figure 6A and Figure S4B). Accordingly, LTPi was prevented by intracellular perfusion of L-NAME via the patch pipette (*p*>0.05, *n* = 11; unpublished data). Moreover, application of the NO donor *S*-nitroso-*N*-acetylpenicillamine (SNAP, 200 µM), in the continuous presence of the phosphodiesterases inhibitor (IBMX, 200 µM), induced an increase of eIPSCs (184.6±32.25 versus 515.3±151.8 pA, before versus after SNAP, respectively; *n* = 9, *p* = 0.039, Wilcoxon signed rank test; Figure 6B and 6C). IBMX was used to prevent nonspecific cGMP degradation [26].

Pharmacological inhibition of the canonical NO receptor guanylylcyclase (GC) with 1H-{1,2,4}oxadiazolo{4,3-a}quinoxalin-&-dione (ODQ, 10 µM) completely blocked the induction of LTPi (control, 206.6±31.36 pA versus 414.6±69.79 pA, before versus after depolarizing steps, *n* = 13, *p* = 0.0064, paired *t* test; ODQ, 261.6±24.01 pA versus 274.4±38.92, before versus after depolarizing steps, *n* = 13, *p* = 0.724, paired *t* test pA; Figure 6D and Figure S4C). Interestingly, when GC activity was impaired by ODQ 5 min after induction of LTPi, its maintenance was preserved (*p*<0.01 Wilcoxon signed rank test; Figure S5A–C), suggesting that constant GC activity is not required for the expression of this form of plasticity.

Protein kinase G (PKG) was shown to be involved in the expression of NO-dependent GABAergic plasticity [27],[28]. Accordingly, when we blocked PKG with the inhibitor KT5823 (500 nM), LTPi was prevented, in fact producing a significant reduction of eIPSCs after the steps (control, 253.6±47.84 pA versus 442.8±66.60 pA, before versus after depolarizing steps, *n* = 11, *p* = 0.007, paired *t* test; KT5828, 213.8±30.31 pA versus 150.8±29.89 pA, before versus after depolarizing steps, *n* = 10, *p* = 0.17, paired *t* test; Figure 6E and Figure S4D).

Importantly, all drugs that were used here to affect various steps of NO signaling did not affect basal GABAergic synaptic transmission (*p*>0.05 in all cases, Figure S5D).

**Figure 7:**
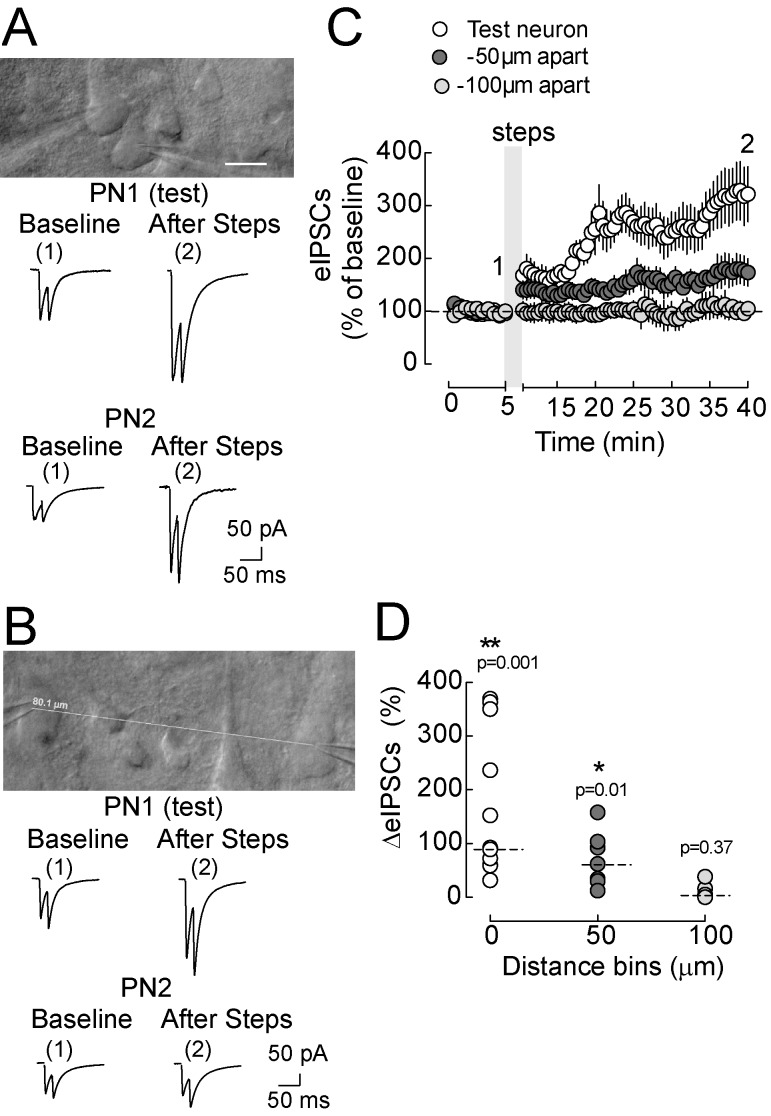
LTPi diffuses to neighboring synapses. (A, Top) Infrared Differential Interference Contrast (IR-DIC) microphotograph of acute cortical slice showing two adjacent recorded layer 5 P neurons (<50 µm). (A, Middle) Representative eIPSC traces of one test (PN1) and one adjacent P neuron (PN2) in control and after injecting depolarizing voltage steps in PN1. IPSCs were evoked in the two neurons by a single stimulating electrode placed nearby their perisomatic region. Note that LTPi was observed also in PN2, despite only PN1 being depolarized. (B) Same experiment of (A) but on a different pair of P neurons recorded at a farther distance (∼80 µm). Note the absence of spread of LTPi in the second, nondepolarized neuron. Scale bar (A and B), 20 µm. (C) Population data of IPSC time course in tested cells (open circles), cells patched up to 50 µm (solid dark gray circles), and at a distance higher than 50 µm from the test neuron (solid light gray circles). (D) ΔeIPSCs of same data of (D). Data are single values and/or mean and median ± SEM. *p<0.05; **p<0.001. Numbers 1 and 2 refer to times of trace illustration.

If NO is involved in LTPi, then it should diffuse to synapses impinging neighboring neurons, as it has been previously shown [29]. We therefore performed simultaneous recordings of two layer 5 pyramidal neurons, separated by various distances. Depolarization of one postsynaptic pyramidal neuron (PN1-test, Figure 7A and 7C) invariably induced a long-term increase of eIPSC amplitudes, as expected (PN1 LTPi amplitude = 170±40.74%, *n* = 11, *p* = 0.001, Wilcoxon signed-rank test). Interestingly, a significant, persistent increase of GABAergic transmission was also observed on a second, unperturbed cell (PN2, Figure 7A–C) if it was within 50 µm from the depolarized cell (PN2 LTPi = 70±19.28%, *n* = 7, *p* = 0.0156, Wilcoxon signed-rank test). In contrast, when the second pyramidal neuron was farther than 50 µm from PN1 (Figure 7B–C), GABAergic transmission was unaffected by PN1 depolarizations (PN2 LTPi = 10±7.7%, *n* = 5, *p* = 0.375, Wilcoxon signed-rank test). Overall, these results confirm the involvement of NO, which, as a gaseous diffusible messenger, can affect synapses impinging neighboring neurons within the cortical circuit.

**Figure 8:**
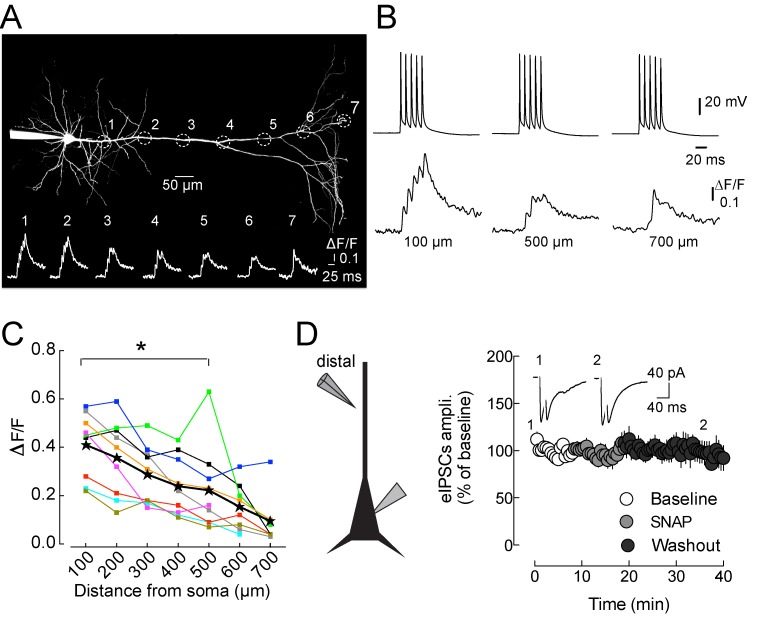
Pyramidal neuron dendritic Ca^2+^ dynamics and lack of NO sensitivity of distal GABAergic synapses. (A) 2PSLM image (maximal intensity projection) of a layer 5 pyramidal neuron loaded with Alexa 594 (20 µM) and the Ca^2+^ indicator OGB-5N (300 µM). Circles and their diameter indicate dendritic recording locations (∼100 µm apart) and approximate dendritic length over which Ca^2+^ transients (in response to 5 APs at 100 Hz) were measured. Inset illustrates representative Ca^2+^ transients (average of 10 trials each) recorded from locations indicated in (A). (B) Representative traces of somatically recorded APs (Top) and corresponding calcium transients recorded at different distances from soma. Note the clear calcium spike recorded at 700 µm from soma. (C) Summary plot showing the peak ΔF/F of bAP-induced Ca^2+^ transients for individual cells as a function of distance from the soma. Each color represents one cell, and stars represent population average values. (D) Time course of dendritic IPSCs recorded from layer 5 pyramidal neurons in the presence of the NO donor SNAP (200 µM for 10 min) and the continuous presence of the nonselective phosphodiesterase inhibitor IBMX (200 µM) and glutamate receptor antagonist DNQX (10 µM). IPSCs were evoked by stimulating GABAergic afferents on pyramidal neuron distal dendrites (∼500 µm from the soma). White, light grey, and grey symbols refer to control, SNAP, and washout periods, respectively. The inset illustrates representative IPSC traces at the two indicated time points (1 and 2).

We have shown that postsynaptic activity does not potentiate dendritic inhibition (Figure 3). To investigate whether the absence of LTPi at dendritic GABAergic synapses was due to a decrease in the dendritic [Ca^2+^] produced by back-propagating APs (bAPs) at distal synapses, we performed two-photon Ca^2+^ imaging while delivering LTPi-inducing bursts of APs. Using the low-affinity calcium indicator OGB-5N, we found that the peak intracellular [Ca^2+^] transient produced by a train of 5 bAPs at 100 Hz decreased along the pyramidal neuron apical dendrite, but not to zero. At ∼500 µm from the soma, corresponding to the location of distal stimulations, the peak change in [Ca^2+^] was still 50% of that in the proximal dendrite (ΔF/F 100 µm, 0.41±0.04 versus ΔF/F 500 µm, 0.22±0.06, *n* = 9, *p* = 0.027, Wilcoxon matched pairs signed rank test; Figure 8A–C). However, if the 50% smaller dendritic [Ca^2+^] transient at distal dendrites is the major cause for the lack of LTPi, increasing dendritic [Ca^2+^] might reveal LTPi at distal synapses. We therefore depolarized pyramidal neurons in voltage clamp, in the presence of intracellular cesium to block K^+^ channels, a condition that permits robust depolarization of distal dendrites [30]. We observed LTPi of perisomatic but not dendritic GABAergic synapses (Figure S6A–B), suggesting that the lack of LTPi at distal inhibitory synapses is not due to reduced Ca^2+^ entry in distal dendrites, but due to a difference downstream.

We considered whether dendrite-targeting interneurons forming distal dendritic GABAergic synapses might lack sensitivity to NO. Therefore, we applied the NO donor SNAP (200 µM, in the continuous presence of the phosphodiesterases inhibitor IBMX, 200 µM) while stimulating dendritic IPSCs that were isolated pharmacologically. We found that, in contrast to perisomatic IPSCs (Figure 6B,C), dendritic GABAergic responses were insensitive to NO (IPSC amplitudes, 100±39.37 and 73.05±17.05 pA; before and 20 min after SNAP application; *n* = 7, *p* = 0.58, Wilcoxon signed rank test; Figure 8D).

To test whether NO-mediated signaling changes GABAergic strength via alteration of presynaptic excitability or alterations in the presynaptic AP waveform of PV cells [5],[31],[32], we tested whether somatic excitability and presynaptic Ca^2+^ dynamics are altered in response to the NO donor SNAP. LTPi induction did not alter resting membrane potential, membrane resistance, firing dynamics, nor somatic AP waveform (*p*>0.05 in all cases; Figure S7A–G). Yet somatic and axonal APs can result from substantially different ion channels. If the terminal AP waveform is changed by NO, this should be reflected by an altered magnitude of Ca^2+^ entry into the presynaptic bouton. However, two-photon imaging of single AP-evoked presynaptic [Ca^2+^] transients in PV-cell boutons, did not reveal NO-dependent alterations in their amplitude (*p*<0.05; Figure S7H–K). These experiments, in addition to LTPi-mediated increase of mIPSC frequency (recorded in TTX), suggest that the expression of LTPi is downstream of Ca^2+^ entry, rather than a mechanism mediated by changes in PV-cell excitability.

Altogether, these data indicate that LTPi depends on retrograde NO signaling, which increases GABA release onto depolarized and nearby pyramidal neurons through a GC-dependent PKG activation. Moreover, the lack of dendritic LTPi is due to lack of NO sensitivity of dendrite-targeting interneurons and not failure to intracellular Ca^2+^ propagation in distal dendrites.

### Modulation of Pyramidal Neuron Synaptic Integration by LTPi

**Figure 9:**
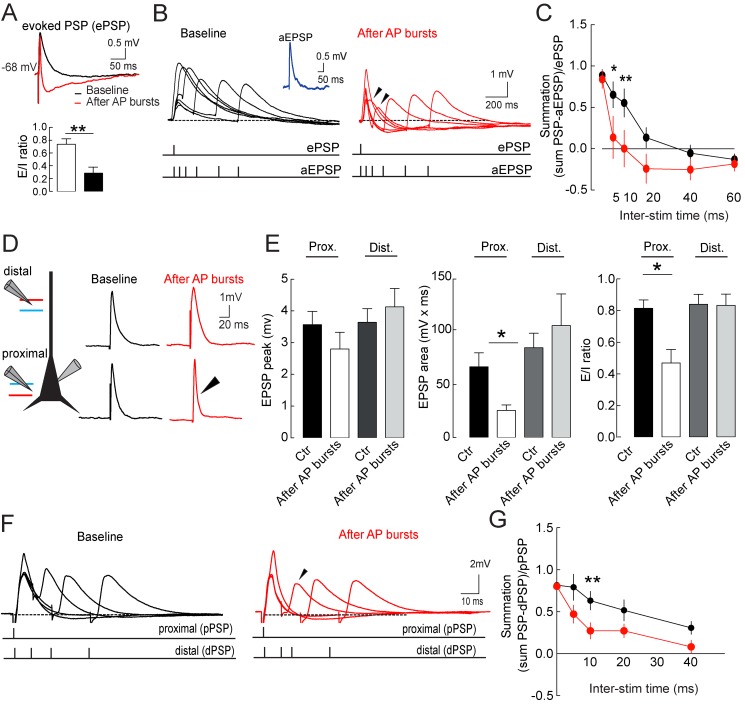
Modulation of pyramidal neuron synaptic integration by LTPi. (A, Top) Representative overlapped traces of evoked EPSP/IPSP sequences (ePSP) in the absence of glutamatergic and GABAergic antagonists, before (black) and 15 min after induction of LTPi (red). LTPi was induced by postsynaptic AP bursts (5 APs at 100 Hz). (A, Bottom) Population E/I ratio analysis before and 15 min after AP burst firing. Data are represented as mean ± SEM. **p<0.01. (B) Representative superimposed traces of aEPSPs summating to proximally evoked PSP at different time intervals before (black traces, Left) and 15 min after LTPi induction (red traces, Right). Arrowheads point to time intervals where the narrowing of the integration window is more evident. The inset illustrates a representative trace (blue) of recorded aEPSP elicited by somatic injection of an aEPSC. (C) Graph illustrating averaged aEPSP-ePSP summation before (black) and 15 min after LTPi induction (red). (D, Left) Schematic of the recording and stimulation configuration. Blue and red lines refer to inhibitory and excitatory afferents, respectively. (D, Right) Composite PSPs before (black traces, Left) and after (red traces, Right) induction of LTPi by AP bursts. Note change of waveform of proximal composite PSP (arrowhead). (E) Graphs showing average depolarizing peaks, areas, and EPSC/IPSC ratio of proximal and distal composite PSPs. No changes were observed in excitatory peak amplitude before and after LTPi. However, a significant reduction of PSP area and EPSC/IPSC ratio was present at proximal PSP selectively. (F) Examples of distal to proximal PSP summation at different time intervals before (Left, black traces) and 15 min after (Right, red traces) induction of LTPi. Arrowhead points to LTPi-dependent change of PSP waveform. (G) Graph illustrating averaged distal to proximal PSP summation before (black) and 15 min after LTPi induction (red). Data are single values mean ± SEM. *p<0.05; **p<0.001. Additional data and analyses are present in Figures S4 and S5.

LTPi-inducing protocols failed to induce long-term plasticity of glutamatergic excitatory synapses (Figure 1D,E), suggesting that LTPi-induced alterations of E/I ratio might modulate the computational properties of pyramidal neurons. Therefore, in current-clamp mode, with physiological intracellular chloride and leaving excitation intact, we evoked EPSP-IPSP sequences (composite PSPs, Figure 9A, top panel). LTPi-inducing burst firing produced a significant change in the composite PSP waveform. Overall, the peak of the depolarizing (EPSP) component was unchanged (baseline, 2.1±0.19 mV; after AP bursts, 1.9±0.22 mV, *n* = 11, *p* = 0.0615, paired *t* test; Figure S8A–B), but the PSP area significantly decreased as a consequence of potentiation of the hyperpolarizing (IPSP) component (baseline, 51.36±8.1 mV/ms; after AP bursts, 19.30±6.1 mV/ms, *n* = 11, *p* = 0.0017, paired *t* test; Figure 9A, Figure S8A–B). Interestingly, however, in some cases, LTPi led to the complete disappearance of the excitatory portion of the composite synaptic response (Figure S8A, example 2). Importantly, LTPi strongly reduced the E/I ratio, measured as the EPSP area divided by the total composite PSP area (baseline, 0.74±0.08; after AP bursts, 0.29±0.09, *n* = 11, *p* = 0.0020, Wilcoxon matched pairs signed rank test; Figure 9A, bottom panel).

To measure synaptic integration we then injected postsynaptic pyramidal neurons with artificial excitatory postsynaptic currents (aEPSCs), producing artificial (a)EPSPs (Figure 9B, inset). Using this approach, we could measure synaptic integration of temporally controlled, fixed-amplitude synaptic events [33]. Indeed, aEPSCs were injected at different intervals from the recorded composite evoked PSPs (Figure 9B). When aEPSPs and composite PSPs occurred simultaneously (time zero), they summated similarly before and after induction of LTPi (normalized synaptic summation, 0.79±0.085 versus 0.67±0.139, baseline versus after AP bursts; *n* = 12, q = 0.1195, F_(11,10)_ = 8.9, *p*>0.05, one-way ANOVA followed by Bonferroni's multiple comparison test; Figure 9B). Interestingly, however, a significant narrowing of the integration window was observed, after LTPi induction, at 5–10 ms time intervals (normalized summation at 5 ms, 0.7±0.16 versus 0.14±0.26, baseline versus after AP bursts; normalized summation at 10 ms, 0.55±0.17 versus 0.001±0.2219, baseline versus after AP bursts; *n* = 12, q = 3.087, *F*_(11,10)_ = 8.9, *p*<0.05 and *p*<0.01 for 5 and 10 ms, respectively, one-way ANOVA followed by Bonferroni's multiple comparison test; Figure 9B).

We reasoned that, because distal GABAergic synapses do not express LTPi (Figure 3), activation of distal inputs can be reliably used to measure synaptic integration, before and after potentiation of perisomatic inhibition. We evoked dendritic and perisomatic synaptic responses in the same pyramidal neuron, by stimulating distal and proximal afferents, respectively (Figure 9D). Separate activation of these two pathways was confirmed by the lack of short-term plasticity, when they were activated in voltage clamp at brief time intervals (Figure S8C–E). Also in these experiments, LTPi altered proximal PSP waveform (*p* = 0.3187 for PSP peak and *p*<0.05 for PSP areas, before and after LTPi induction; Kruskal–Wallis test; *n* = 12; Figure 9D). No significant changes were observed at distal PSP before and after LTPi induction (*p*>0.05; *n* = 12; Figure 9D–E).

Moreover, the E/I ratio decreased at proximal synapses after LTP induction, but it was unaltered at distal synapses (*p*<0.05 baseline versus after bursts for proximal stimulation, and *p*>0.05 for distal stimulation; Kruskal–Wallis test, *n* = 12; Figure 9E, right panel). Even in this case, LTPi did not alter summation at time zero (F_(9,84)_ = 5.116, *p*>0.05 one-way ANOVA followed by Bonferroni's multiple comparison test; Figure 9F–G), but PSP summation was significantly reduced at 8–12 ms intervals following potentiation of GABAergic synapses (normalized summation, 0.65±0.16 versus 0.14±0.26, baseline versus AP bursts, respectively, q = 3.997, F_(9,84)_ = 5.116, *p*<0.01, one-way ANOVA followed by Bonferroni's multiple comparison test, *n* = 12; Figure 9F–G). Overall, these experiments indicate that layer 5 pyramidal neurons can alter their ability of summating temporally dispersed synaptic events in response to self-induced potentiation of GABAergic proximal synapses.

### Long-Term Plasticity of GABAergic Synapses Strongly Affects Pyramidal Neuron Spike Output

**Figure 10:**
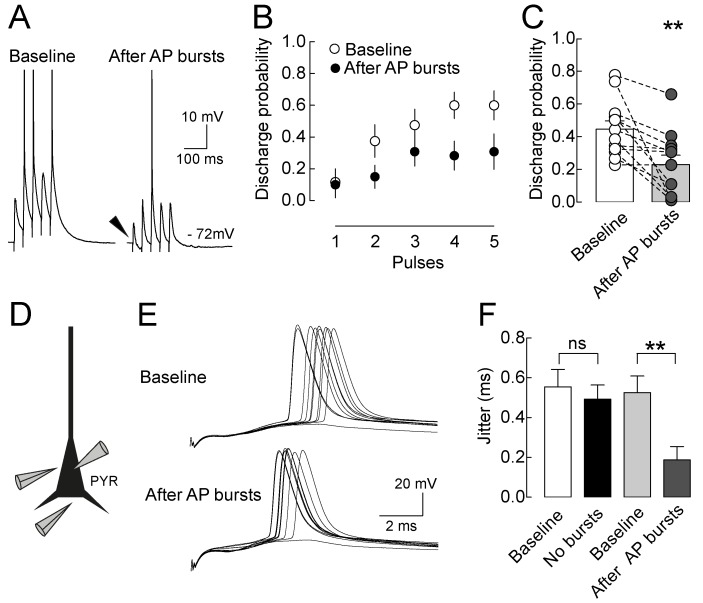
Long-term plasticity of GABAergic synapses strongly affects pyramidal neuron spike output. (A) Representative voltage recordings of a layer 5 pyramidal neuron showing PSP summation and occasional AP firing in response to a presynaptic train of 5 pulses at 25 Hz before (left trace) and after LTPi induction (right trace). Note the change in the first PSP waveform (arrowhead) and the reduced ability of firing after LTPi. APs were truncated for illustration purposes. (B) Graph illustrating average pyramidal neuron discharge probability before (open circles) and 15 min after LTPi (solid circles) for each synaptic stimulus within the train. (C) Population graph illustrating the total discharge probability across the entire train. (D) Schematic drawing of the recording and stimulating configuration: a layer 5 pyramidal neuron is recorded, while PSCs are simultaneously evoked by two stimulating electrodes placed near the perisomatic region. (E) Representative voltage traces showing AP firing in response to synaptic afferent stimulation before (Top) and after (Bottom) inducing LTPi by intracellularly evoked AP bursts. Note that after LTPi induction, APs occur with higher temporal precision. (F) Population data of AP jitter calculated at two time points (10 and 30 min) in the absence (white and black columns) and presence (light and dark grey columns) of LTPi-inducing bursts. Note that a significant reduction of spike jitter (i.e., increased temporal precision) occurs only after LTPi induction. Data are single mean ± SEM. **p<0.001.

How does this alteration of synaptic integration window translate into spike output of layer 5 pyramidal neurons? Perisomatic E/I ratio is strongly reduced after LTPi induction, thereby likely contributing to a modification in the spike probability of pyramidal neurons. To test this hypothesis, we stimulated perisomatic synaptic afferents to layer 5 pyramidal neurons in short trains (5 pulses at 25 Hz). Stimulation intensity was adjusted in order to evoke sporadic firing as a result of EPSP summation (Figure 10A). Spike probability was calculated as the number of APs divided by the number of trials at each individual stimulus. We found that the spike probability dramatically decreased after intracellularly evoked, LTPi-inducing AP bursts (5 APs at 25 Hz, repeated 10 times every 1.5 s; spike probability, 0.45±0.05 versus 0.23±0.06, control versus LTPi, respectively; *n* = 12, *p* = 0.0043, paired *t* test; Figure 10B–C). The presence of LTPi was confirmed as a change of composite PSP waveform (as in Figure 9A,C and Figure 10A). Interestingly, decrease of discharge probability was absent in a subset of cells that did not express LTPi (spike probability, 0.42±0.03 versus 0.46±0.02, baseline versus after bursts; *n* = 4, *p* = 0.25 Wicoxon signed rank test). Importantly, EPSP trains were evoked at the same membrane potentials, before and after LTPi induction.

Because GABAergic transmission was shown to modulate the precision of synaptically evoked APs [34], we then tested if LTPi alters the timing of synaptically evoked spikes. Suprathreshold responses were evoked by simultaneous stimulations of layer 5 pyramidal neurons' perisomatic afferents (Figure 10D). Stimulus strength was adjusted to induce >50% AP firing, to prevent complete loss of spikes in response to LTPi. We found that LTPi largely decreased the AP jitter, measured as the standard deviation of spike times (0.52±0.083 versus 0.19±0.06 ms, baseline versus 20 min after LTPi induction; *n* = 8, *p*<0.01, one-way ANOVA followed by Bonferroni's multiple comparison test; Figure 10E–F). Importantly, control experiments performed in the absence of LTPi-inducing bursts failed to change synaptically induced spike precision (0.55±0.087 versus 0.49±0.072 ms, baseline versus 20 min after LTPi induction; *n* = 8, *p*>0.05, one-way ANOVA followed by Bonferroni's multiple comparison test; Figure 10E–F).

These results indicate that layer 5 pyramidal neuron activity can selectively potentiate perisomatic inhibition, thereby reducing the ability of generating spikes but strongly improving their temporal precision.

## Discussion

We found that neocortical layer 5 pyramidal neurons augment perisomatic GABAergic transmission over long timescales (LTPi), in response to increases in their electrical activity (non-associative). Strikingly, we found that LTPi-inducing stimuli selectively potentiated perisomatic GABAergic synapses from PV basket cells, resulting in a decrease of E/I ratio, which altered synaptic integration, reduced firing probability, and increased spike-time precision of layer 5 principal cells. LTPi required postsynaptic intracellular Ca^2+^ elevation through L-type Ca^2+^ channels, triggering NO retrograde signaling, which by acting on a GC- and PKG-dependent mechanism increases GABA release.

Previously, Kurotani et al. [22] showed that postsynaptic activity (albeit at hyperpolarized potentials) can induce LTP of GABAergic synapses onto layer 5 pyramidal neurons, through altered trafficking of postsynaptic GABA_A_Rs. However, the plasticity of GABAergic synapses that we report here is fundamentally different as it does not require strong hyperpolarization (up to −90 mV [22]), and it relies on increased presynaptic release of neurotransmitter, induced by retrograde NO signaling. In our hands, we could not potentiate postsynaptic GABA_A_R functionality, measured by perisomatic GABA uncaging, in response to stimuli that induce LTPi. In addition, we never observed LTD of GABAergic transmission at postsynaptic membrane potentials ranging between −60 and −70 mV, as reported by Kurotani et al. [22]. The GABA plasticity we describe here well agrees with the non-Hebbian potentiation that was very recently described at GABAergic thalamic synapses [23]. The apparent discrepancy with Kurotani et al. [22] could be due to intrinsic differences in pyramidal neuron populations in different sensory cortices (somatosensory versus visual). In addition, we cannot exclude that pre- and postsynaptic expression of GABAergic plasticity could be induced depending on the neuronal state, preferential innervation by specific interneuron classes [35], and/or specific firing patterns of pyramidal neurons. It is interesting to consider that various forms of plasticity can coexist, depending on the actual correlated activation of various neuron types during specific cortical network activities. In any case, we demonstrate here that single pyramidal neurons can auto-modulate the strength of afferent GABAergic synapses but not of glutamatergic afferents, in response to their own firing activity.

This LTPi was found in ∼72% of recorded layer 5 pyramidal neurons and varied in magnitude. This variability is similar to that observed in other studies (e.g., [22],[28]), but we cannot rule out the possibility that the recorded neurons exhibit a potentiation that is dependent on the amount of previous activity and hence on the initial plastic state just prior to induction protocols. Alternatively, experimental variability could arise from whole-cell dialysis or a combination of several biological processes, including (*i*) variability in postsynaptic Ca^2+^ increases, (*ii*) heterogeneous enzymatic activity and NO mobilization, (*iii*) differential amount of NO production, and (*iv*) presence of tonic GC activity at some presynaptic terminals.

### Cellular Mechanism of LTPi

What is the mechanism underlying LTPi in layer 5 pyramidal neurons? Our experiments indicate that NO is involved as a potential retrograde messenger, produced postsynaptically and acting at presynaptic GABAergic terminals. Here we provide several lines of evidence that support this interpretation: (*i*) neuronal NO synthase (nNOS) is often expressed postsynaptically and requires intracellular Ca^2+^elevations [36]–[38]; (*ii*) pharmacological blockade of NOS as well as disruption of the GC-sensitive, PKG pathway completely prevented LTPi [27],[28]; (*iii*) LTPi could be mimicked by a NO donor; and (*iv*) LTPi diffuses to GABAergic synapses, impinging neighboring nonstimulated pyramidal neurons at distances that were compatible with previously described NO-dependent plasticity of glutamatergic synapses in hippocampus and cerebellum [29],[39],[40].

Analyses of CV, IPSC PPR, and mIPSC frequency before and after LTPi induction suggest that the expression of LTPi is presynaptic and likely alters release probability (Figure 4) [5],[31],[32]. We confirmed the presynaptic locus by showing that pIPSCs are not altered after LTPi induction. We did not detect a difference in AP-induced Ca^2+^ transients recorded from single basket cell boutons before and after NO donor application, suggesting that augmentation of release cannot be due to alterations in presynaptic spike waveform or Ca^2+^ entry. We favor the hypothesis that PKG activation by cGMP alters the synaptic vesicle machinery, thus changing the probability of GABA release, as LTPi was associated with increase of mIPSC frequency in TTX and there was no effect of SNAP on PV-cell excitability and presynaptic Ca^2+^ dynamics.

Anatomical data suggest that nNOS is expressed selectively by small subpopulations of GABAergic neurons [41], although several lines of evidence indicate that both nNOS mRNA [42] and protein [43] can be present in neocortical layer 5 pyramidal neurons. We cannot exclude, however, that NO is produced by other NOS isoforms and/or cellular elements present in the neuropil, including nonneuronal cells [44]. Although expression of nNOs by pyramidal neurons is controversial, it is noteworthy that several forms of glutamatergic LTP rely on NO production, likely from pyramidal neurons [45]–[47]. In any case, here we demonstrate that postsynaptic Ca^2+^-dependent activity alone results in presynaptic alterations via an NO-dependent retrograde signaling.

It will be fundamental to reveal in future studies what minimal firing activity and/or Ca^2+^ levels are required to induce LTPi. Likewise, it will be important to decipher the molecular pathways underlying its maintenance. Here we show that impairment of GC activity after LTPi induction did not prevent the maintenance of GABAergic plasticity. This indicates that LTPi involves sequential activation of soluble CG, cGMP-dependent protein kinase, and possibly cGMP-degrading phosphodiesterase [48]. This cycle might be important in maintaining a critical cGMP level necessary to sustain LTPi [48].

### Selective Potentiation of GABAergic Synapses Originates from Specific Interneuron Types

LTPi expression is likely restricted to perisomatic-targeting GABAergic synapses, as indicated by potentiation of IPSCs originating from FS, PV basket cells (either in paired recordings or optogenetic experiments) by pyramidal neuron depolarization. Moreover, no changes in synaptic strength were observed when evoking GABAergic inhibition at more distal regions and when dendrite-targeting SST interneurons were selectively stimulated optogenetically. One possible explanation for lack of LTPi expression at GABAergic dendritic synapses is an insufficient increase in dendritic [Ca^2+^] by bAPs [49]. Indeed, [Ca^2+^] elevations at 500 µm (location of distal synaptic stimulation) were half the size of those measured from the proximal dendritic compartments. Nevertheless, LTPi was absent in voltage clamp, with intracellular Cs^+^, a condition favoring the dendritic spread of depolarization [30]. Moreover, the selective lack of effect by the NO donor SNAP on distal GABAergic responses indicates that the absence of LTPi at dendritic synapses can be largely explained by an insensitivity of dendrite-targeting interneurons to NO. Remarkably, in line with our experiments, neocortical expression of the NO receptor GC seems to be preferentially expressed by perisomatic targeting PV basket cells [50].

### Compartment-Selective “Unlocking” of E/I Ratio Via LTPi

One major finding of this study is that AP firing and/or depolarization inducing LTPi did not alter glutamatergic synaptic transmission, although NO-dependent forms of glutamatergic plasticity have been reported [29],[45]–[47]. The activity-dependent self-regulation of GABAergic synapses reported here altered the E/I ratio onto layer 5 pyramidal neurons. Whereas changes in E/I balance have been commonly associated with pathological states [3], here we show that this equilibrium can be altered at the level of single pyramidal neurons, and in an interneuron-selective manner, in response to their physiological firing. Indeed, LTPi was induced by short postsynaptic bursts of APs, commonly observed in layer 5 pyramidal neurons *in vivo* spontaneously [12] and in response to sensory stimuli [13]. It will be interesting to test if LTPi can, at least in part, support the hypothesis that short AP bursts can be optimal encoders of sensory information [51]. Indeed, top-down, feedback sensory inputs in distal dendrites increase the tendency of pyramidal neurons to fire in bursts [52],[53]. Could LTPi of perisomatic inhibition regulate the processing of feed-forward sensory information thought to arrive more proximally in the dendrites?

Whereas the persistent strengthening of glutamatergic synapses has been proposed to have a key role in development and memory [54],[55], the role of plasticity of GABAergic synapses is currently largely unknown. LTPi can powerfully modulate the impact of synaptic inputs targeting specific pyramidal neuron compartments. Importantly, perisomatic inhibition is fundamental for network synchronization during cortical oscillations [14],[56]. Therefore, a persistent increase of perisomatic inhibition, with unaltered excitation, will have profound effects on the computational properties of cortical principal neurons. Indeed, precision of cortical neuron firing and their ability to act as coincident detectors is governed by how excitatory inputs are curtailed by inhibition [33],[57]. Here we show that selective strengthening of perisomatic GABAergic synapses narrowed the time window for integration of temporally dispersed excitatory inputs. Consequently, in response to LTPi, layer 5 pyramidal neurons tend to fire significantly less, but with a much improved temporal precision. Thus, it will be interesting to test whether selective potentiation of perisomatic inhibition onto single pyramidal neurons changes their specific temporal association to global cortical network activity, likely affecting the relay of information to other cortical and subcortical areas.

In addition, the perisomatic specificity of LTPi is of great importance if one considers that in layer 5 pyramidal neurons somatic activity increases the distal dendritic computation of incoming information [52]. Hence, selective plasticity of perisomatic inhibition could alter the way sensory information is perceived in distal dendrites, in addition to its role shown here to modulate pyramidal neuron spike output.

## Materials and Methods

### Animals

Experimental procedures followed national (French and Italian) and European guidelines, and have been approved by the authors' institutional review boards. Experiments were done on C57BL/6 wild-type mice. In some experiments, to identify GABAergic transmission from PV and SST interneurons, we used PV-Cre and SST-cre mice (Jackson Laboratory stock nos. 008069 and 013044, respectively [19]). To selectively express EGFP in PV-positive cells, we bred PV:Cre with RCE:loxP (kindly provided by Gordon Fishell, New York University) or td-Tomato:loxP reporter mice (Jackson stock no. 007908) obtaining PV-Cre::RCE [18] or PV-Cre::td-Tomato mice.

### *In Vitro* Slice Preparation and Electrophysiology

Parasagittal slices (320 µm thick) from somatosensory cortex were obtained from 15- to 28-d-old mice. Animals were deeply anesthetized with isofluorane and decapitated. Brains were quickly removed and immersed in “cutting” solution (4°C) containing the following (in mM): 126 choline, 11 glucose, 26 NaHCO_3_, 2.5 KCl, 1.25 NaH_2_PO_4_, 10 MgSO_4_, 0.5 CaCl_2_, 3 pyruvic acid, 3 myo-inositol, and 0.4 ascorbic acid (equilibrated with 95% O_2_/5% CO_2_). Slices were cut with a vibratome (Leica) in cutting solution and then incubated in oxygenated artificial cerebrospinal fluid (ASCF) containing the following (in mM): 125 NaCl, 2.5 KCl, 2 CaCl_2_, 1 MgSO_4_, 1.25 mM NaH_2_PO_4_, 26 mM NaHCO_3_, and 16 mM glucose (pH 7.4), initially at 34°C for 30 min, and subsequently at room temperature, before being transferred to the recording chamber. Recordings were obtained at 30°C. Synaptic currents were recorded in whole-cell voltage- or current-clamp mode of layer 5 pyramidal neurons of mouse primary barrel somatosensory cortex visually identified using infrared video microscopy by their large somata and pia-oriented apical dendrites. For voltage-clamp experiments, electrodes (with a tip resistance of 2–4 MΩ) were filled with an intracellular solution containing (in mM): 70 K-gluconate, 70 KCl, 10 Hepes, 0.2 EGTA, 2 MgCl_2_, 4 MgATP, 0.3 MgGTP, 5 Na-phosphocreatine, 0.05 QX314-Cl, pH adjusted to 7.2 with KOH, 280–300 mOsm. The estimated *E*_Cl_ was approximately −16 mV based on the Nernst equation, without correction for gluconate-generated liquid junction potential. Under these recording conditions, activation of GABA_A_ receptors resulted in inward currents at a holding potential (*V*_h_) of −70 mV. In experiments with a cesium-based solution, electrodes (Figure S6) were filled with an intracellular solution containing (in mM): 70 CsMeSO3, 70 CsCl, 10 Hepes, 0.2 EGTA, 2 MgCl_2_, 4 MgATP, 0.3 MgGTP, 5 Na-phosphocreatine, 0.05 QX314-Cl, pH adjusted to 7.2 with CsOH, 280–300 mOsm. In current-clamp experiments, electrodes were filled with an intracellular solution containing (in mM): 135 K-gluconate, 10 KCl, 10 Hepes, 0.2 EGTA, 2 MgCl_2_, 4 MgATP, 0.3 MgGTP, 5 Na-phosphocreatine, pH adjusted to 7.2 with KOH, 280–300 mOsm. In order to isolate GABA_A_-receptor-mediated currents, DNQX (10 µM) was present in the superfusate of all experiments, unless otherwise indicated. GABA_A_-receptor-mediated currents were evoked via a glass microelectrode filled with ACSF positioned in the perisomatic region of the pyramidal cell (see Figures 3 and 9). In experiments in which perisomatic and dendritic inhibition was evoked, we called distal synapses those stimulated in layer 2/3 within the same column of the recorded layer 5 pyramidal neuron. Synaptic responses were evoked every 3 s (0.33 Hz) in voltage-clamp experiments and every 10 s (0.1 Hz) in current-clamp mode. In experiments including postsynaptic calcium buffer, 20 mM K-gluconate were replaced by 20 mM BAPTA and 2 mM Ca^2+^ was added. In experiments where EPSCs were evoked, the GABA_A_R antagonist gabazine (10 µM) was added to the ACFS.

Signals were amplified, using a Multiclamp 700B patch-clamp amplifier (Axon Instruments, Foster City, CA), sampled at 20 kHz and filtered at 4 kHz or 10 kHz. Data were analyzed using pClamp (Axon Instruments), IGOR PRO 5.0 (Wavemetrics), and GraphPad Prism software. Custom-written software (detector, courtesy of J. R. Huguenard, Stanford University) was used to analyze spontaneous and miniature GABAergic events. All drugs were obtained from Tocris Cookson (Bristol, UK) or Sigma or Ascent Scientific (Bristol, UK).

In voltage-clamp experiments, access resistance was on average <20 MΩ and monitored throughout the experiment. Recordings were discarded from analysis if the resistance changed by >20% over the course of the experiment. In current-clamp experiments, input resistance was monitored with small current steps (−25 pA for 400 ms) and cells were excluded if it changed by >25%.

For paired recordings between pyramidal neurons and PV interneurons, these latter cells were identified as expressing EGFP in PV-Cre::RCE mice. Importantly, all EGFP-expressing interneurons showed FS firing behavior in response to depolarizing current steps [9]. Presynaptic PV interneurons were recorded using an intracellular solution containing (in mM): 130 K-gluconate, 10 KCl, 10 Hepes, 0.2 EGTA, 2 MgCl_2_, 4 MgATP, 0.3 MgGTP, 5 Na-phosphocreatine, pH adjusted to 7.2 with KOH, 280–300 mOsm. The estimated *E*_Cl_ was approximately −58 mV. Unitary synaptic responses were elicited in voltage-clamp mode by brief somatic depolarizing steps evoking action currents in presynaptic PV cells.

aEPSCs were generated in MATLAB (MathWorks), using the following equation:
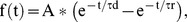
where τd and τr are the decay and rise time constants respectively, and A is a constant related to aEPSC amplitude. Typically, τd and τr had values of 0.5 and 3 ms, respectively; A was adjusted in every cell to yield aEPSP amplitudes similar to the “test” extracellularly evoked PSPs.

In experiments detailed in Figure 8, the peak of proximal and distal EPSPs were binned in 3-ms intervals, as often EPSP peaks were not coincident, due to differences in rise times and/or latencies.

LTPi was induced in voltage clamp by 10 depolarizing steps to 0 mV (from a holding of −70 mV) lasting 5 s and delivered every 30 s. In current-clamp experiments detailed in Figures 8A–C, 9, and 10, LTPi was induced with 15 bursts of 5 APs at 100 Hz, delivered every 10 s.

### Virus-Mediated Gene Delivery and Optogenetics

To selectively express the light-sensitive ion channel channelrhodopsin 2 (ChR2) in SST- or PV-expressing cortical interneurons, SST- or PV-Cre pups (P1–2) were anesthetized on ice, and a beveled injection pipette, attached to a micromanipulator, was gently inserted 300 µm deep in the somatosensory cortex through intact skin and skull. We then delivered 300 nL of viral particles (in PBS) using an injector (Nanoliter 2000 Injector, WPI Inc., USA), and the pipette was left in place for an additional 30 s, before it was retracted. The adeno-associated viral (AAV) particles expressed floxed ChR2 (AAV9.EF1.dflox.hChR2(H134R)-mCherry.WPRE.hGH; Addgene 20297) and were obtained from the Penn Vector Core (University of Pennsylvania). At the end of the procedure, pups were returned to their mother until P15–28, when they were sacrificed to obtain slices for electrophysiological experiments, as described above. ChR2 activation was obtained by brief (ranging between 0.5 and 2 ms) light flashes on cortical slices, using a 5W LED (λ = 470 nm, Thorlabs) collimated and coupled to the epifluorescence path of a Zeiss AxioExaminer microscope. Experiments were performed using a 60× water immersion lens. Light-evoked responses were recorded in layer 5 pyramidal neurons and were almost completely abolished by gabazine (10 µM; Figures S2 and S3).

### Immunofluorescence

Slices used for electrophysiology experiments were fixed overnight in 4% paraformaldehyde in phosphate buffer (PB, pH 7.4) at 4°C. Slices were then rinsed three times at room temperature (10 min each time) in PB and incubated overnight at 4°C in PB with 0.3% Triton X-1000, 0.1% normal donkey serum (NDS), and primary rabbit anti-SST antibody (1∶200, Peninsula Lab. Inc./Bachem). Slices were then rinsed three times in PB (10 min each) at room temperature and incubated with Cy-2–anti-rabbit antibody (1∶400; Jackson IR) for 3.5 h at room temperature. Slices were then rinsed three times in PB (10 min each) at room temperature and coverslipped in mounting medium. Immunofluorescence was then observed with a confocal microscope (Leica) and images were acquired.

### Two-Photon Imaging

Layer 5 pyramidal neurons in somatosensory cortex were identified and whole-cell patched using infrared Dodt contrast (Luigs and Neumann, Ratingen, Germany) and a frame transfer CCD camera (Scion Corporation, Cairn Research Ltd, Faversham, UK) Two-photon fluorescence imaging was performed with a femtosecond pulse Ti:Sapphire laser (Cameleon Ultra II, Coherent) tuned to 810 nm coupled into an Ultima laser scanning head (Prairie Technologies, Middleton, WI), mounted on an Olympus BX61W1 microscope, and equipped with a water-immersion objective (60×, 1.1 numerical aperture, Olympus Optical, Tokyo, Japan). Pyramidal cell morphology was visualized using fluorescence imaging of patch-loaded Alexa 594 (20 µM). Dendritic Ca^2+^ transients were recorded using 300 µM of the calcium indicator OGB-5N and using rapid line scan imaging (∼10 µm at 0.956 ms per line). Lines were drawn by hand, either along several microns of dendritic length or, in the case of presynaptic imaging, perpendicular to the longitudinal axis of the dendrite.

For presynaptic Ca^2+^ imaging, PV-positive interneurons, identified as expressing td-Tomato in PV-Cre::td-Tomato mice, were loaded, via a whole-cell pipette, with 200 µM Fluo-5F and 20 µM Alexa Fluor 594 in a solution containing (mM): 110 KMeSO_3_, 40 HEPES, 10 Na-phosphocreatine, 4 MgCl_2_, 4 Na_2_-ATP, 0.4 Na GTP, 0.01 EGTA. The dyes were allowed to equilibrate for at least 50 min before recording [Ca^2+^] transients. “Green” and “red” fluorescence was separated from excitation wavelengths using a long pass dichroic (660dcxr) followed by a spectral beam splitter (575dcxr) and barrier filters for the green and red channels (hq525/70m-2p and hq607/45m-2p, respectively). All filters were purchased from Chroma (USA). Fluorescence was detected using both proximal epifluorescence and substage photomultiplier tubes: multi-alkali (R3896, Hamamatsu, Japan) and gallium arsenide phosphide (H7422PA-40 SEL, Hamamatsu) for the red and green channels, respectively.

### Analysis

Time-dependent Ca^2+^ fluorescence transients were constructed from line scan images by spatial averaging the fluorescence over those pixels corresponding to dendritic lengths, or width of the bouton, resulting in a single fluorescence trace as a function of time. The background fluorescence was estimated from the average pixel intensity of those pixels not on a labeled structure. This average value was subtracted at all time points of the fluorescence trace. The trace was then converted to ΔF/F(*t*) by subtracting the mean resting fluorescence (averaged over 20 ms just prior to electrical stimulation), then scaling the traces by the same value. Offline filtering was performed using a 2 pt binomial smoothing filter. All fluorescence transient analysis was performed using Neuromatic, running in the Igor Pro environment (Wavemetrics).

### GABA Uncaging

A 488 nm laser was coupled into the photolysis pathway of the Ultima two-photon laser scanning head and then focused to the back focal plane of the objective, resulting in a 5 µm spot. We used 1 ms laser pulses to photolyse the caged compound RUBI-GABA (Tocris Bioscience), which was bath applied at a concentration of 20 µM (in normal ACSF). pIPSCs were evoked every 30 s. 2PSLM images of Alexa 594 (20 µM) were used to position the laser spot on the perisomatic region of whole-cell patched L5 pyramidal neurons.

### Statistical Analysis

Analysis of LTP was performed by comparing the mean amplitude of ePSCs or ePSPs in the last 10 min of the plasticity to the baseline period. Unless otherwise indicated, statistical comparisons were done between values of mean amplitudes.

In the summation experiments (Figure 9) analysis was done as previously described [33]. Normality of the data was assessed (D'Agostino & Pearson omnibus normality test). Normal distributions were statistically compared using paired *t* test two-tailed or one-way ANOVA followed by Bonferroni's Multiple Comparison *post hoc* test to compare several independent groups. When data distributions were not normal or *n* was small (e.g., Figure 3E), nonparametric tests were performed (e.g., Figure 3). When comparing the effect of postsynaptic depolarizations or AP bursts in different groups, changes of eIPSCs (e.g., Figures S1A and 7) were expressed as:
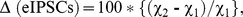
where χ_1_ is the mean of IPSC amplitudes before steps or bursts (10 min of baseline) and χ_2_ is the mean of IPSC amplitudes after steps or bursts (20–30 min after LTPi induction protocol). Therefore, when Δ (eIPSCs) = 0, LTPi-inducing protocols failed to induce changes compared to baseline [58]. We then used the Wilcoxon signed-rank test to compare the relative data with the hypothetical value of 0 (i.e., no effect). When several independent groups were compared, we performed Kruskal–Wallis test followed by Dunn's Multiple Comparison *post hoc* test. Differences were considered significant if *p*<0.05. Values are presented as mean ± SEM of *n* experiments.

## Supporting Information

Figure S1 LTPi of GABAergic synapses onto layer V neurons is majorly expressed presynaptically. (A) Normalized changes of eIPSCs (ΔeIPSCs, see Materials and Methods) in response to postsynaptic depolarizations. Grey symbols and white symbols refer to pyramidal neurons that did and did not express LTPi, respectively. (B, Left) Analysis of the squared coefficients of variations of evoked IPSCs (CV^2^1/CV^2^2) as described by [59]. Numbers 1 and 2 refer to baseline and after depolarization values, respectively. According to this analysis, CV^2^ values on the horizontal line (I) reflect a postsynaptic potentiation, whereas cells in region II (above the diagonal linear fit line) showed a presynaptic LTPi expression. Values in region III refer to P neurons with a mixed pre- and postsynaptic LTPi expression. Overall, apart from a few exceptions, layer 5 P neurons showed a presynaptic locus of LTPi expression. (B, Right) LTPi was accompanied by a reduction in CV (grey bar), whereas cells that were not depolarized had no change in CV (white bar). (C) LTPi was accompanied with a decrease in PPR (grey bars), whereas cells that were not depolarized had a constant PPR overtime. (D, Left) mIPSCs rise-time distribution. No change of rise-time distribution was observed after LTPi-inducing stimuli (red distribution). (D, Right) mIPSC rise times during baseline (white bar and symbols) and after steps (grey bar and black symbols). Note the very fast rise times (<1 ms), indicating that inhibitory quantal events were mostly perisomatic. No change was observed after LTPi induction. Data are represented as mean ± SEM.
(TIF)

Figure S2 Characterization of photo-induced distal IPSCs. (A) Two-photon fluorescence images of parasagittal sections of layer V S1 of an SST-Cre mouse injected with the adeno-associated virus expressing floxed ChR2 (AAV9.EF1.dflox.hChR2(H134R)-mCherry.WPRE.hGH; Addgene 20297). Left, mCherry-labeled infected neurons (red labeling); Middle, SST immunoreactivity (green labeling); Right, merged image reflecting high degree of colocalization between infected neurons and SST labeling. (B) Firing pattern of a mCherry-labeled neuron in response to a 1-s current injection step. Note the typical low-threshold burst typical of SST-positive Martinotti cells [60],[61]. (C) Voltage response to a single (Left) or a train of five 470 nm light pulses (Right), recorded in a mCherry-labeled neuron. Light pulse duration was 2 ms. (D) Representative SST-cell-mediated IPSC recorded in a layer V pyramidal neuron triggered by a 2-ms-long, 470-nm light pulse (black trace). This response was GABAergic, as it was completely abolished by application of 10 µM gabazine (red trace).
(TIF)

Figure S3 LTPi can be reliably induced by optogenetic activation of PV-positive interneurons. (A–B) ChR2 was co-expressed with mCherry in PV-Cre mice using viral vectors (see Materials and Methods). (Right) Brief (0.5–1 ms) flashes of 470 nm light (blue bars) induced GABAergic currents, which reliably potentiated in response to LTPi-inducing protocols (black trace, control; red trace, after postsynaptic depolarizing steps). Intracellular loading of 20 mM BAPTA completely prevented LTPi induction as in Figure 5A–B. (C–D) Photo-stimulated IPSCs in PV and SST cells (black versus red traces) showed different rise times, consistent with the differential perisomatic versus dendritic targeting of pyramidal neurons. **p<0.01. (E) The gabazine-resistant photo-stimulated inward current (red trace) was completely abolished by TTX (0.5 µM).
(TIF)

Figure S4 Single values of Ca^2+^-dependent NO signaling in LTPi. Plots of individual eIPSC amplitudes before (*x*-axes) versus 20 min after postsynaptic depolarizations (*y*-axes) in control experiments (ctr; open circles in all panels) and in conditions where we prevented (A) intracellular Ca^2+^ elevations with 20 mM BAPTA in the patch pipette, (B) activation of CB1Rs with 2 µM AM 251 (Middle) and NOS activity with 100 µM L-NAME, (C) GC activity with 10 µM ODQ, and (D) PKG activity with 500 nM KT5823. These experiments indicate the involvement of Ca^2+^-dependent retrograde NO signaling and exclude the involvement of CB1Rs. NO activates PKG via a GC-dependent mechanism.
(TIF)

Figure S5 LTPi maintenance does not depend on NO signaling. (A–C) Late application of ODQ (black bar in B) did not blunt potentiation of GABAergic responses. (D) Pharmacological perturbation of the NO signaling cascade did not affect basal GABAergic transmission onto layer 5 pyramidal neurons.
(TIF)

Figure S6 LTPi is present at perisomatic but absent at distal inhibitory synapses. (A, Left) Representative voltage-clamp traces of IPSCs evoked by stimulating distal and proximal inhibitory inputs before and 20 min after postsynaptic depolarizing steps. A cesium-based intracellular solution was used in order to block postsynaptic potassium channels and allow further propagation of membrane depolarization along the dendritic tree. (A, Right) Average time courses of proximal (dark grey) and distal (light grey) IPSCs, showing no overall LTPi at GABAergic synapses impinging distal (∼500 µm) apical dendrites. (B) Plots of individual eIPSC amplitudes before (*x*-axes) versus 20 min after postsynaptic depolarizations (*y*-axes) at proximal (Left) and distal (Right) synapses.
(TIF)

Figure S7 LTPi is not associated with changes of excitability of presynaptic PV basket cells. (A–B) AP dynamics of PV basket cells did not change in the presence of the NO donor SNAP. (A) Representative voltage traces from a PV cell in response to a hyperpolarizing (−50 pA) and depolarizing current step (150 pA) before and 20 min after SNAP application. (B) Population data illustrating that the firing rate was unaffected by SNAP over a wide range of depolarizing stimuli. (C) Neither resting membrane potential (Left) nor membrane resistance was affected by the NO donor. (D and E) Overlapped traces of single spikes (D) and their relative phase plots (E) show that single AP waveform was unaffected by SNAP (red trace). Inset in (E) is a blowout of the phase plot corresponding to the grey square to illustrate the criterion used to calculate the spike threshold. (F and G) Population data illustrating lack of SNAP effect on spike threshold (F) and phase plot positive and negative peaks (G). Results in (D–G) indicate that NO did not affect single spike properties of PV cells. (H) 2PSLM image (maximal intensity projection) of a layer 5 PV basket cell loaded with Alexa 594 (20 µM). The neuron was filled with the Ca^2+^ indicator Fluo-5F (200 µM), and Ca^2+^ transients were measured in presynaptic boutons as illustrated in the right panel. (I–K) Representative traces (I) and population data (K) of intraterminal Ca^2+^ transients evoked by single APs fired at the soma in control and 20 min after SNAP application. Fluorescence was stable during the recording time periods as shown in (J).
(TIF)

Figure S8 LTPi alters the E/I ratio onto layer 5 P neurons. (A) Representative current-clamp traces of EPSP-IPSC sequence recorded in low physiological intracellular chloride. Two examples are shown illustrating how the selective potentiation of the GABAergic, hyperpolarizing component of the composite PSP curtailed the glutamatergic, depolarizing part (example 1). This led, in some cases, to the complete disappearance of the EPSP (example 2). (B) Population analysis of the depolarizing component of the composite PSP before and 15 min after AP burst firing. (C) Schematic of the recording and stimulating configuration. (D) Representative voltage-clamp traces of EPSC evoked by stimulations of the same distal (dEPSC, Top) or proximal (pEPSC, Bottom) pathway at brief (20 ms) intervals. Note the presence of paired-pulse facilitation in both cases. (E) When the two pathways were activated at the same interval but independently, no short-term plasticity was present, indicating that the two stimulating electrodes could activate distinct glutamatergic pathways. Data are represented as mean ± SEM. **p<0.01.
(TIF)

## Abbreviations

aEPSP: artificial EPSP
AP: action potential
bAP: back-propagating action potential
BAPTA: 1,2-bis(o-aminophenoxy)ethane-N,N,N′,N′-tetraacetic acid
CB1: cannabinoid receptor type-1
cGMP: cyclic guanosine monophosphate
ChR2: Channelrhodopsin2
CV: coefficient of variation
DNQX: 6,7-dinitroquinoxaline-2,3-dione
EGTA: ethylene glycol tetraacetic acid
E/I: excitation to inhibition ratio
eEPSC: evoked excitatory postsynaptic current
eEPSP: evoked excitatory postsynaptic potential
eIPSC: evoked IPSC
eIPSP: evoked inhibitory postsynaptic potential
FS: fast-spiking
GABA: gamma aminobutyric acid
GC: guanylyl cyclase
IBMX: 3-isobutyl-1-methylxanthine
IPSC: inhibitory postsynaptic current
L-NAME: L-N^G^-Nitroarginine methyl ester
LTDi: long-term depression of inhibition
LTPi: long-term potentiation of inhibition
mIPSC: miniature IPSC
NMDA: N-methyl-D-aspartate
nNOS: neuronal nitric oxide synthase
NO: nitric oxide
ODQ: 1H-[1,2,4]oxadiazolo[4,3-a]quinoxalin-1-one
OGB: Oregon Green BAPTA
pIPSC: photolysis-evoked IPSC
PKG: protein kinase G
PN: pyramidal neuron
PPR: paired-pulse ratio
PV: parvalbumin
sIPSC: spontaneous IPSC
SNAP: (±)-S-Nitroso-N- acetylpenicillamine
SST: somatostatin
TTX: tetrodotoxin
uIPSC: unitary IPSC
VGCC: voltage-gated calcium channel
