# Supplementary figures and images for: Non-associative Potentiation of Perisomatic Inhibition Alters the Temporal Coding of Neocortical Layer 5 Pyramidal Neurons
Source: PLoS Biol. 2014 Jul 8;12(7):e1001903. doi: 10.1371/journal.pbio.1001903 (PMC4086817; doi:10.1371/journal.pbio.1001903)

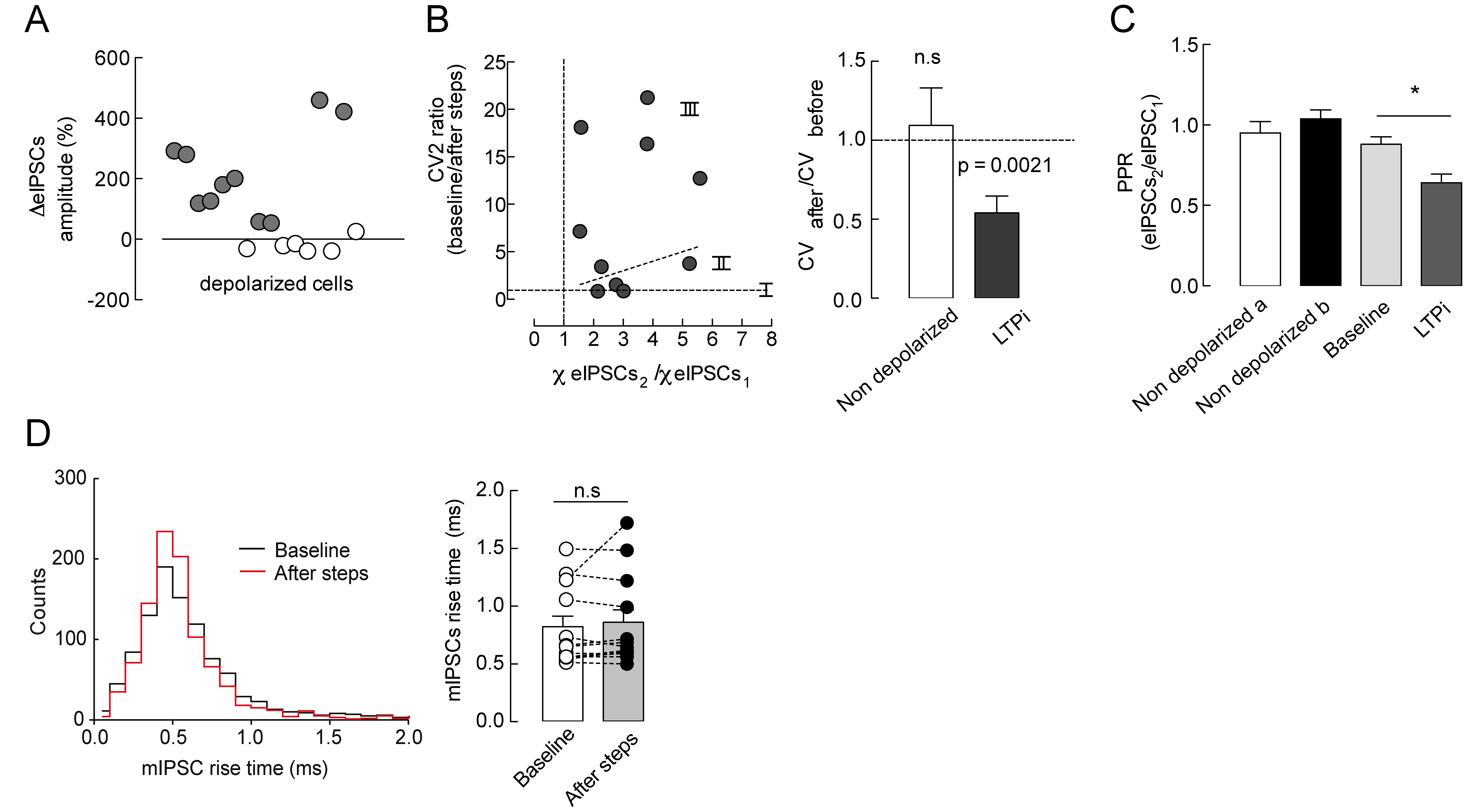

Supplement: Figure S1 — LTPi of GABAergic synapses onto layer V neurons is majorly expressed presynaptically. (A) Normalized changes of eIPSCs (ΔeIPSCs, see Materials and Methods) in response to postsynaptic depolarizations. Grey symbols and white symbols refer to pyramidal neurons that did and did not express LTPi, respectively. (B, Left) Analysis of the squared coefficients of variations of evoked IPSCs (CV21/CV22) as described by [59]. Numbers 1 and 2 refer to baseline and after depolarization values, respectively. According to this analysis, CV2 values on the horizontal line (I) reflect a postsynaptic potentiation, whereas cells in region II (above the diagonal linear fit line) showed a presynaptic LTPi expression. Values in region III refer to P neurons with a mixed pre- and postsynaptic LTPi expression. Overall, apart from a few exceptions, layer 5 P neurons showed a presynaptic locus of LTPi expression. (B, Right) LTPi was accompanied by a reduction in CV (grey bar), whereas cells that were not depolarized had no change in CV (white bar). (C) LTPi was accompanied with a decrease in PPR (grey bars), whereas cells that were not depolarized had a constant PPR overtime. (D, Left) mIPSCs rise-time distribution. No change of rise-time distribution was observed after LTPi-inducing stimuli (red distribution). (D, Right) mIPSC rise times during baseline (white bar and symbols) and after steps (grey bar and black symbols). Note the very fast rise times (<1 ms), indicating that inhibitory quantal events were mostly perisomatic. No change was observed after LTPi induction. Data are represented as mean ± SEM. (TIF) [file pbio.1001903.s001.tif]

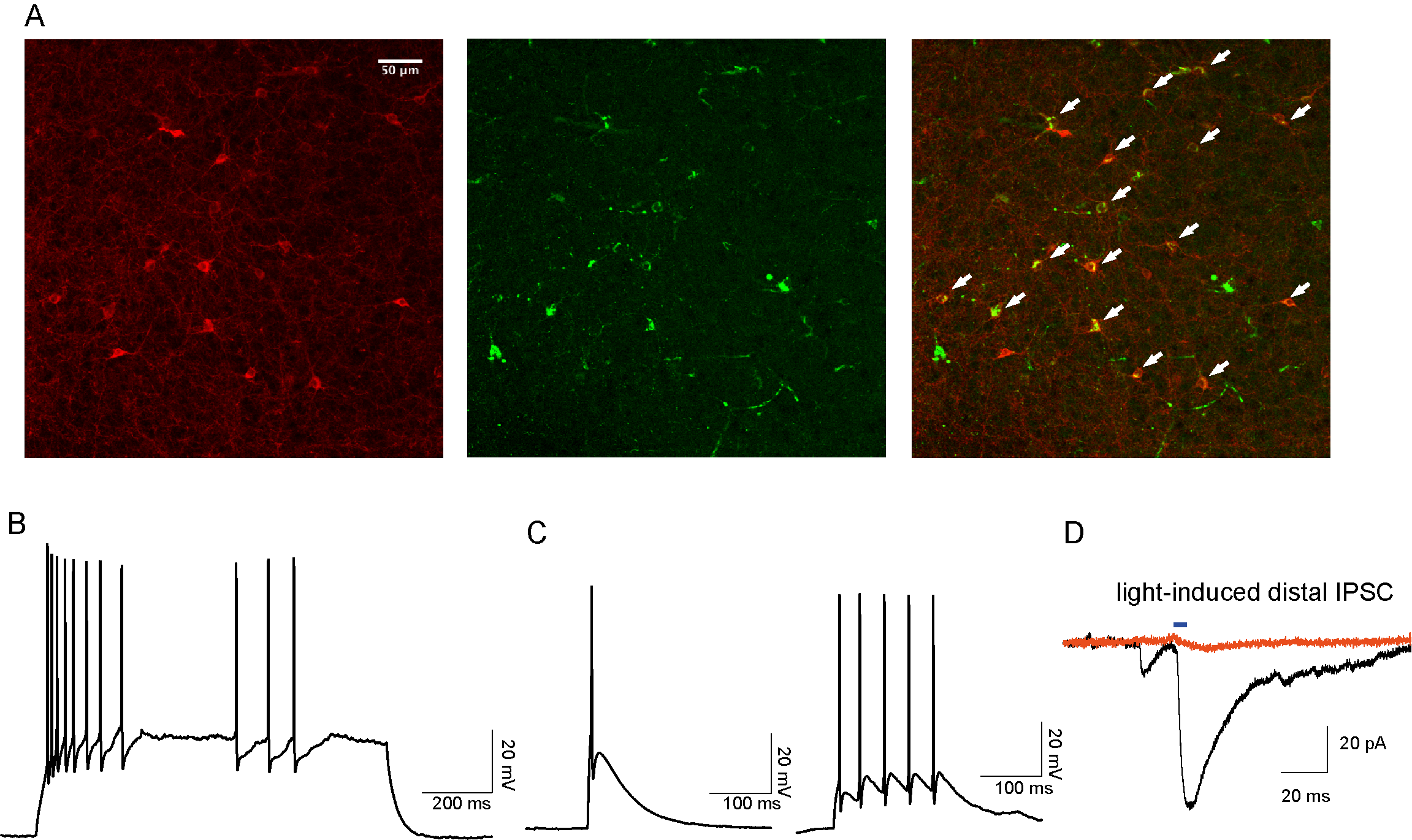

Supplement: Figure S2 — Characterization of photo-induced distal IPSCs. (A) Two-photon fluorescence images of parasagittal sections of layer V S1 of an SST-Cre mouse injected with the adeno-associated virus expressing floxed ChR2 (AAV9.EF1.dflox.hChR2(H134R)-mCherry.WPRE.hGH; Addgene 20297). Left, mCherry-labeled infected neurons (red labeling); Middle, SST immunoreactivity (green labeling); Right, merged image reflecting high degree of colocalization between infected neurons and SST labeling. (B) Firing pattern of a mCherry-labeled neuron in response to a 1-s current injection step. Note the typical low-threshold burst typical of SST-positive Martinotti cells [60],[61]. (C) Voltage response to a single (Left) or a train of five 470 nm light pulses (Right), recorded in a mCherry-labeled neuron. Light pulse duration was 2 ms. (D) Representative SST-cell-mediated IPSC recorded in a layer V pyramidal neuron triggered by a 2-ms-long, 470-nm light pulse (black trace). This response was GABAergic, as it was completely abolished by application of 10 µM gabazine (red trace). (TIF) [file pbio.1001903.s002.tif]

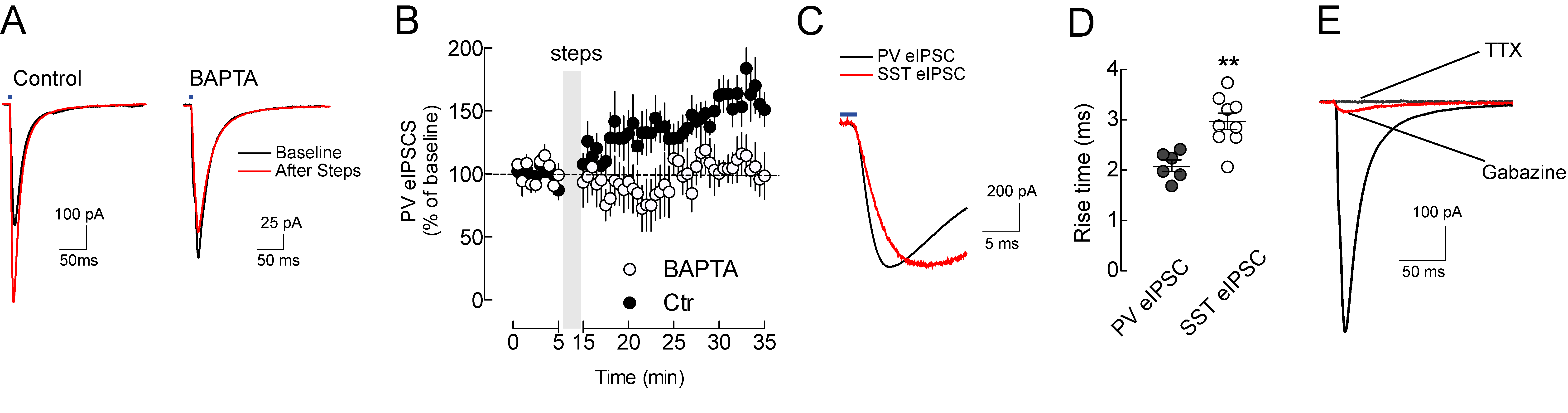

Supplement: Figure S3 — LTPi can be reliably induced by optogenetic activation of PV-positive interneurons. (A–B) ChR2 was co-expressed with mCherry in PV-Cre mice using viral vectors (see Materials and Methods). (Right) Brief (0.5–1 ms) flashes of 470 nm light (blue bars) induced GABAergic currents, which reliably potentiated in response to LTPi-inducing protocols (black trace, control; red trace, after postsynaptic depolarizing steps). Intracellular loading of 20 mM BAPTA completely prevented LTPi induction as in Figure 5A–B. (C–D) Photo-stimulated IPSCs in PV and SST cells (black versus red traces) showed different rise times, consistent with the differential perisomatic versus dendritic targeting of pyramidal neurons. **p<0.01. (E) The gabazine-resistant photo-stimulated inward current (red trace) was completely abolished by TTX (0.5 µM). (TIF) [file pbio.1001903.s003.tif]

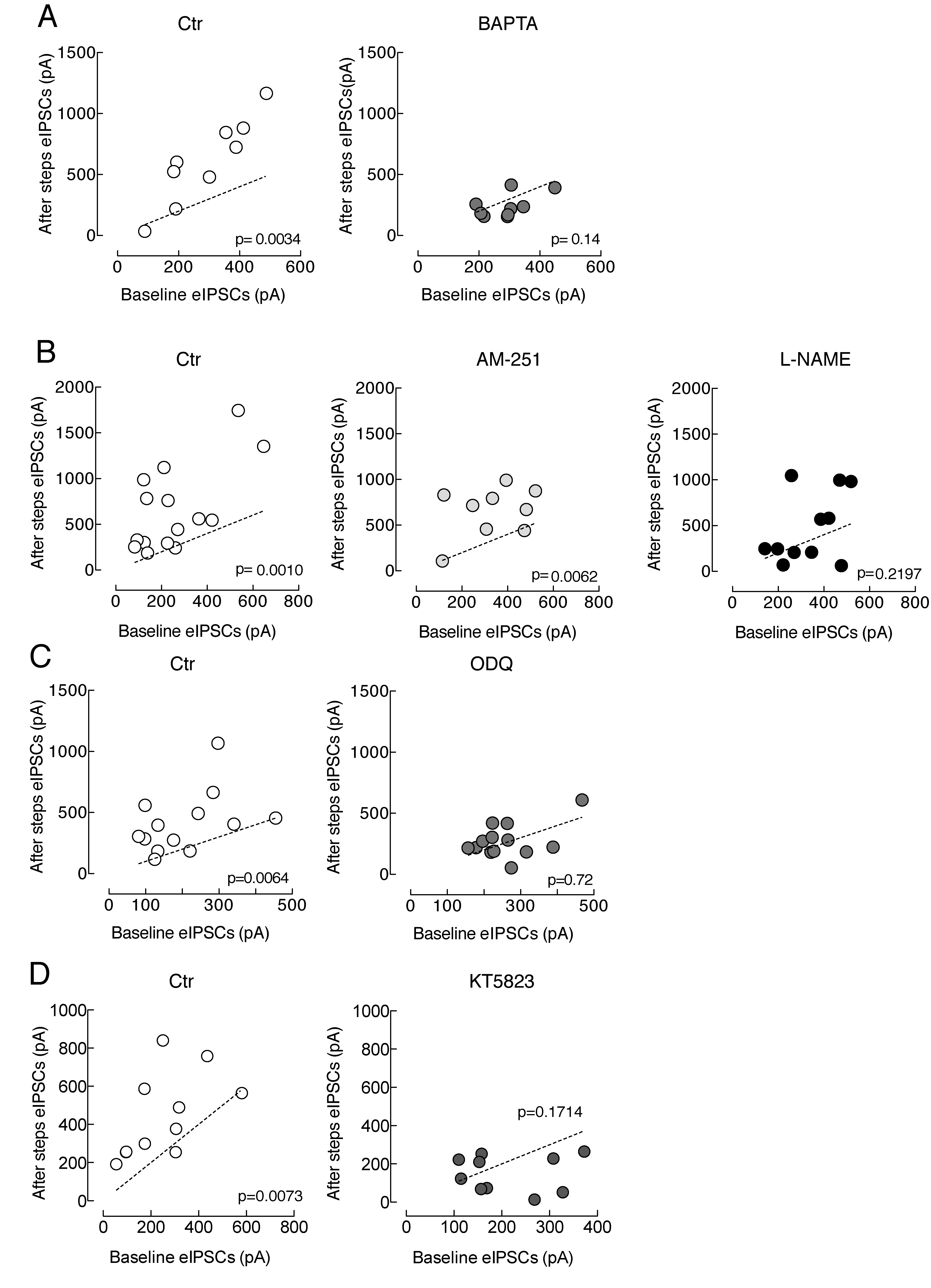

Supplement: Figure S4 — Single values of Ca2+-dependent NO signaling in LTPi. Plots of individual eIPSC amplitudes before (x-axes) versus 20 min after postsynaptic depolarizations (y-axes) in control experiments (ctr; open circles in all panels) and in conditions where we prevented (A) intracellular Ca2+ elevations with 20 mM BAPTA in the patch pipette, (B) activation of CB1Rs with 2 µM AM 251 (Middle) and NOS activity with 100 µM L-NAME, (C) GC activity with 10 µM ODQ, and (D) PKG activity with 500 nM KT5823. These experiments indicate the involvement of Ca2+-dependent retrograde NO signaling and exclude the involvement of CB1Rs. NO activates PKG via a GC-dependent mechanism. (TIF) [file pbio.1001903.s004.tif]

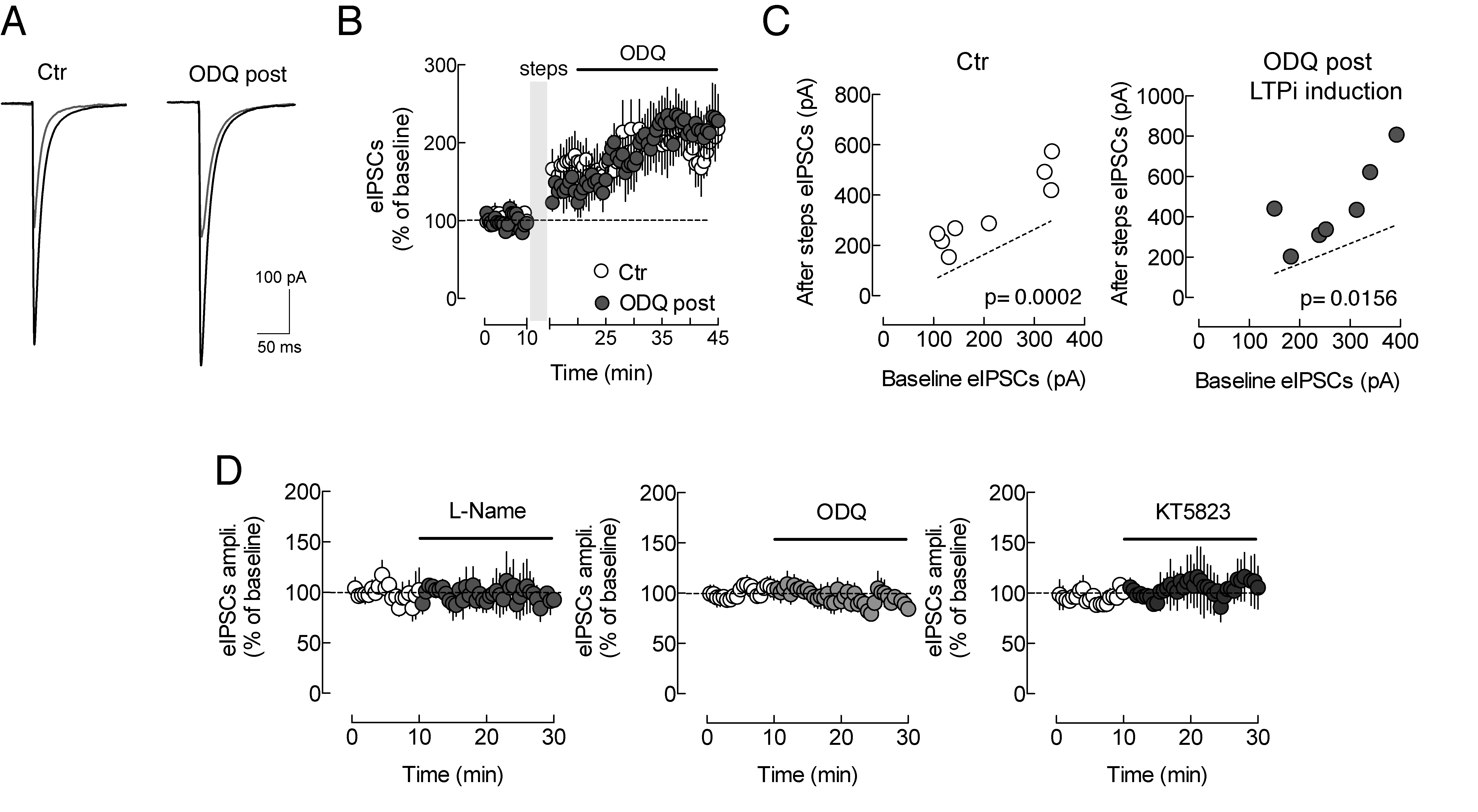

Supplement: Figure S5 — LTPi maintenance does not depend on NO signaling. (A–C) Late application of ODQ (black bar in B) did not blunt potentiation of GABAergic responses. (D) Pharmacological perturbation of the NO signaling cascade did not affect basal GABAergic transmission onto layer 5 pyramidal neurons. (TIF) [file pbio.1001903.s005.tif]

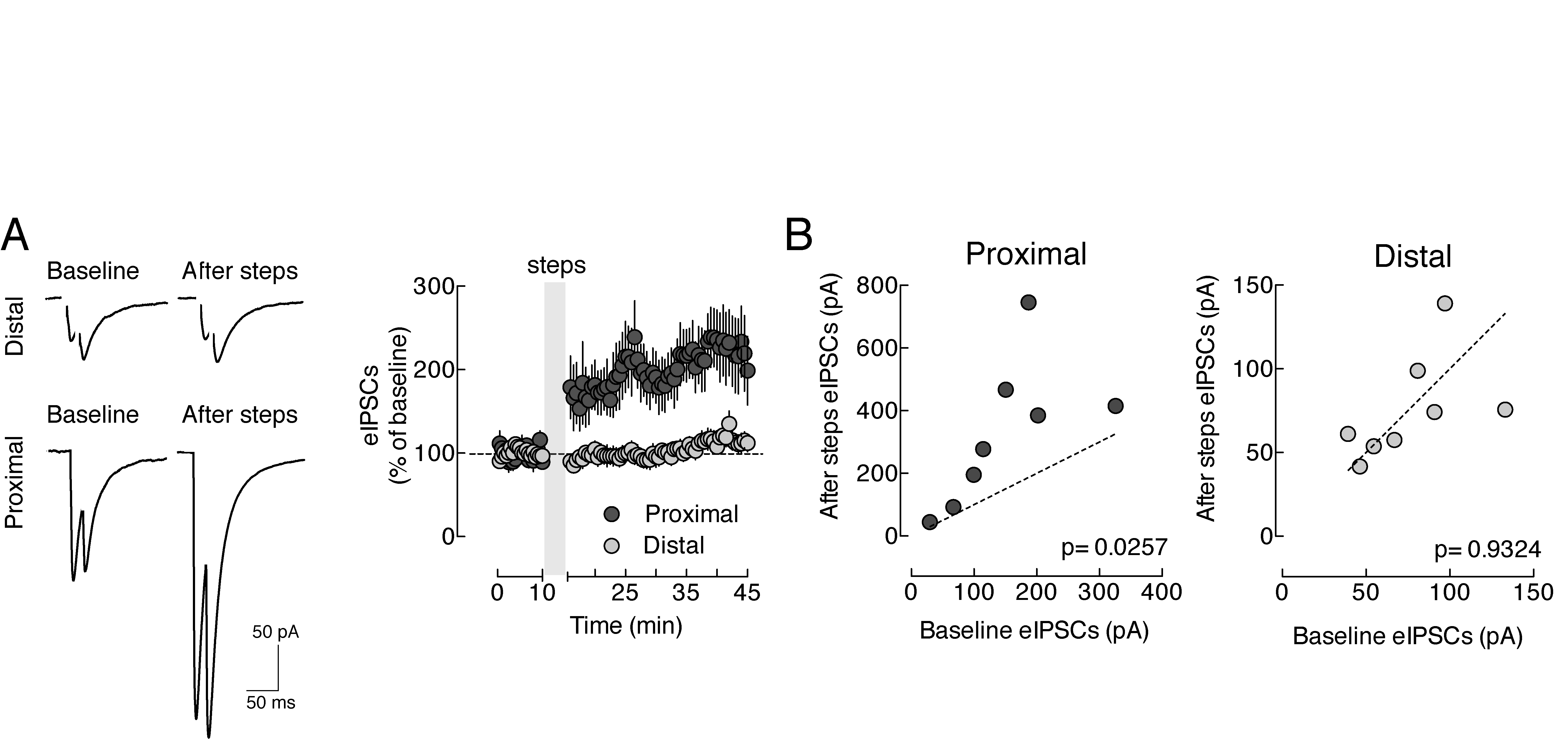

Supplement: Figure S6 — LTPi is present at perisomatic but absent at distal inhibitory synapses. (A, Left) Representative voltage-clamp traces of IPSCs evoked by stimulating distal and proximal inhibitory inputs before and 20 min after postsynaptic depolarizing steps. A cesium-based intracellular solution was used in order to block postsynaptic potassium channels and allow further propagation of membrane depolarization along the dendritic tree. (A, Right) Average time courses of proximal (dark grey) and distal (light grey) IPSCs, showing no overall LTPi at GABAergic synapses impinging distal (∼500 µm) apical dendrites. (B) Plots of individual eIPSC amplitudes before (x-axes) versus 20 min after postsynaptic depolarizations (y-axes) at proximal (Left) and distal (Right) synapses. (TIF) [file pbio.1001903.s006.tif]

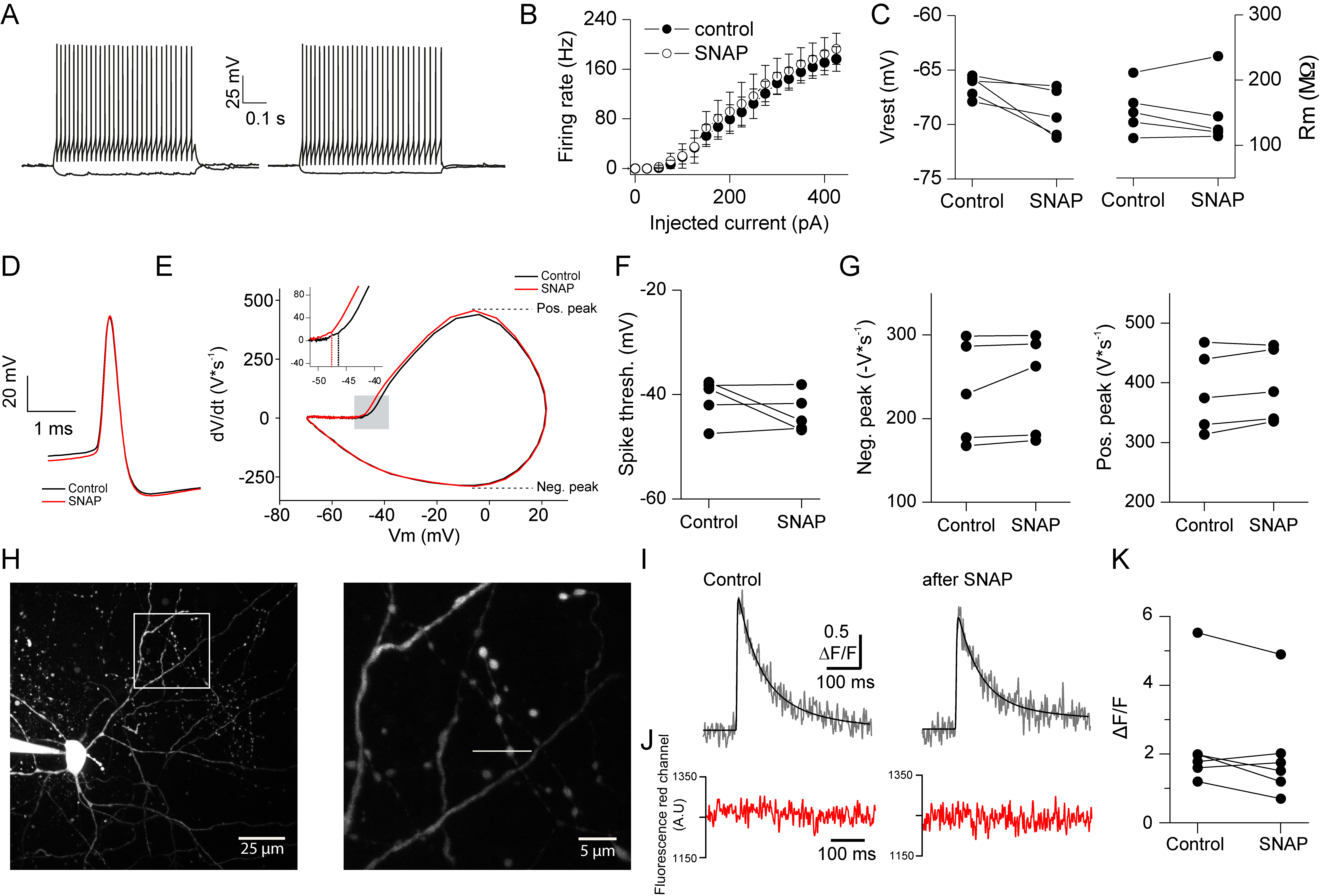

Supplement: Figure S7 — LTPi is not associated with changes of excitability of presynaptic PV basket cells. (A–B) AP dynamics of PV basket cells did not change in the presence of the NO donor SNAP. (A) Representative voltage traces from a PV cell in response to a hyperpolarizing (−50 pA) and depolarizing current step (150 pA) before and 20 min after SNAP application. (B) Population data illustrating that the firing rate was unaffected by SNAP over a wide range of depolarizing stimuli. (C) Neither resting membrane potential (Left) nor membrane resistance was affected by the NO donor. (D and E) Overlapped traces of single spikes (D) and their relative phase plots (E) show that single AP waveform was unaffected by SNAP (red trace). Inset in (E) is a blowout of the phase plot corresponding to the grey square to illustrate the criterion used to calculate the spike threshold. (F and G) Population data illustrating lack of SNAP effect on spike threshold (F) and phase plot positive and negative peaks (G). Results in (D–G) indicate that NO did not affect single spike properties of PV cells. (H) 2PSLM image (maximal intensity projection) of a layer 5 PV basket cell loaded with Alexa 594 (20 µM). The neuron was filled with the Ca2+ indicator Fluo-5F (200 µM), and Ca2+ transients were measured in presynaptic boutons as illustrated in the right panel. (I–K) Representative traces (I) and population data (K) of intraterminal Ca2+ transients evoked by single APs fired at the soma in control and 20 min after SNAP application. Fluorescence was stable during the recording time periods as shown in (J). (TIF) [file pbio.1001903.s007.tif]

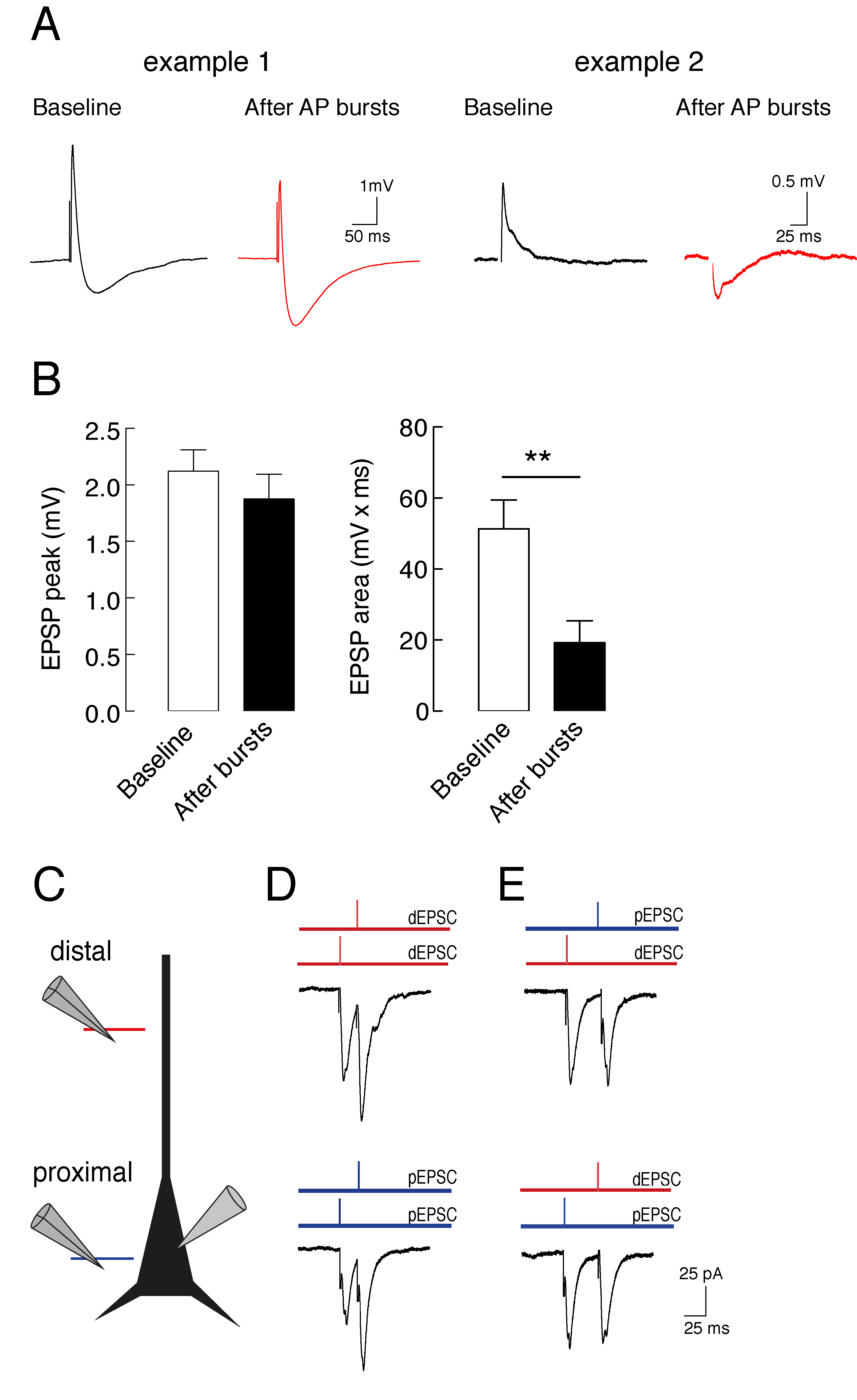

Supplement: Figure S8 — LTPi alters the E/I ratio onto layer 5 P neurons. (A) Representative current-clamp traces of EPSP-IPSC sequence recorded in low physiological intracellular chloride. Two examples are shown illustrating how the selective potentiation of the GABAergic, hyperpolarizing component of the composite PSP curtailed the glutamatergic, depolarizing part (example 1). This led, in some cases, to the complete disappearance of the EPSP (example 2). (B) Population analysis of the depolarizing component of the composite PSP before and 15 min after AP burst firing. (C) Schematic of the recording and stimulating configuration. (D) Representative voltage-clamp traces of EPSC evoked by stimulations of the same distal (dEPSC, Top) or proximal (pEPSC, Bottom) pathway at brief (20 ms) intervals. Note the presence of paired-pulse facilitation in both cases. (E) When the two pathways were activated at the same interval but independently, no short-term plasticity was present, indicating that the two stimulating electrodes could activate distinct glutamatergic pathways. Data are represented as mean ± SEM. **p<0.01. (TIF) [file pbio.1001903.s008.tif]
